# Fatty Acids in Cancer Therapy: Chemical Conjugates, Nanocarriers, and Therapeutic Opportunities

**DOI:** 10.3390/molecules31111848

**Published:** 2026-05-27

**Authors:** Gabriela Antal, Nicoleta Anamaria Pașcalău, Elisabeta Atyim, Oana Bătrîna, Codruța Șoica, Marius Mioc, Cristina Tandafirescu, Alexandra Mioc

**Affiliations:** 1 Faculty of Pharmacy, “Victor Babes” University of Medicine and Pharmacy, Eftimie Murgu Square, No. 2, 300041 Timișoara, Romania; gabriela.antal@umft.ro (G.A.); elisabeta.atyim@umft.ro (E.A.); oana.esanu@umft.ro (O.B.); codrutasoica@umft.ro (C.Ș.); marius.mioc@umft.ro (M.M.); trandafirescu.cristina@umft.ro (C.T.); alexandra.mioc@umft.ro (A.M.); 2 Research Center for Experimental Pharmacology and Drug Design (X-Pharm Design), “Victor Babes” University of Medicine and Pharmacy, Eftimie Murgu Square, No. 2, 300041 Timișoara, Romania; 3 Department of Psycho Neuroscience and Recovery, Faculty of Medicine and Pharmacy, University of Oradea, 410087 Oradea, Romania

**Keywords:** fatty acids, anticancer, oncology, FA-conjugation, lipid prodrug, derivatization, lipid-based formulations, esterification, liposomes, lipid nanoparticles

## Abstract

Fatty acids (FAs) have drawn attention in the field of oncology due to their multifaceted role, not only as structural components of lipid-based delivery systems but also as functional moieties that can enhance the pharmacokinetic and biological behavior of anticancer drugs and, subsequently, their therapeutic performance. Due to their biocompatibility, structural diversity, high affinity for biological membranes, and albumin-binding capacity, FAs can increase drug lipophilicity, membrane permeability, systemic distribution, tissue distribution, and enable controlled enzymatic release. All these properties endorse the development of nanocarriers containing FAs, such as liposomes, lipid nanoparticles (LNPs), self-nanoemulsifying drug delivery systems (SNEDDS), and self-assembling lipidic prodrugs (LAPs). In addition, several FAs, especially polyunsaturated FAs, seem to have a direct anticancer activity by modulating lipid metabolism, oxidative stress, membrane organization, and regulating cell death pathways. This review summarizes the FA conjugation chemistry, the influence of FA on the pharmacokinetics and tumor-targeting capacity of anticancer agents, and the current developments in FA-based cancer treatment strategies, while also covering the biological functions of FA in cell death pathways and cancer metabolism. By integrating medicinal chemistry, nanocarrier design, pharmacokinetic modulation, and tumor lipid biology, this review positions FA-based strategies as a relevant and evolving platform for improving anticancer drug delivery, tumor selectivity, and therapeutic performance.

## 1. Introduction

Cancer is a serious and, unfortunately, very common pathology among people nowadays. Numerous researchers, doctors and pharmacists alike, carry out continuous and intense research work to find the most effective treatments in oncology, with minimal side effects. Despite major advances achieved in recent years in chemotherapy, immunotherapy and targeted therapies, many anticancer compounds still present significant pharmacological limitations. Presently addressed by numerous researchers, the persistent challenges that must be overcome include suboptimal pharmacokinetics, poor tumor selectivity, dose-limiting toxicity, limited therapeutic index or rapid systemic clearance. Addressing these matters by improving drug delivery, biodistribution, and ensuring a controlled release remains a central objective to enhance therapeutic efficacy of anticancer compounds, while minimizing their adverse effects.

**Figure 1:**
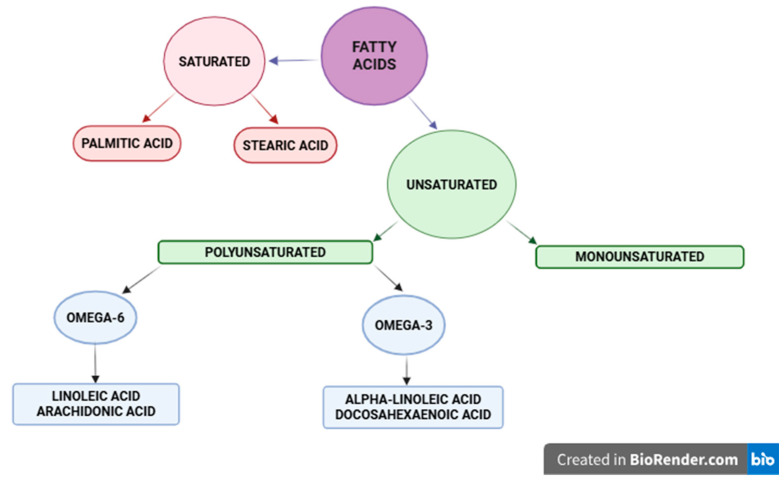
Classification of FAs. Based on the type of the covalent bond, FAs can be subdivided into: (a) saturated (SFA—with no double bond) and (b) unsaturated (UFA—with at least one double bond); the unsaturated FA can be further subdivided into: (i) monounsaturated (MUFA—with one double bond) and (ii) polyunsaturated (PUFA—with two or more double bonds). Created in BioRender. Antal, G. (2026) https://BioRender.com/nzsdpif.

In this context, lipid-based strategies have gained increasing attention as promising strategies to overcome these limitations. Lipids are naturally occurring biomolecules with high biocompatibility and structural diversity that play central roles in cellular physiology and have the capacity to interact with biological membranes. Among lipid classes, FA, important components of phospholipids, cholesterol esters and triglycerides, are essential for cellular metabolism and play key roles in cell signaling pathways and gene expression, cell membrane structure and function, and energy production [1,2]. Chemically, FA structure differs from lipids containing steroid and isoprenoid scaffolds; FAs contain hydrocarbon chains of variable sizes displaying a methyl group (-CH_3_) at one end of the molecule and a carboxyl group (-COOH) at the other end. FA classification is presented in Figure 1.

The evolution of FAs in biomedical research and oncology has significantly evolved from passive nutritional components to active therapeutic agents that can be used as multifunctional elements in drug design and delivery [3].

The role of fatty acids in biomedical research has advanced from their early identification as important structural and metabolic elements of biological membranes to their present-day use as versatile components in drug design and nanomedicine. Initial research predominantly examined the nutritional, metabolic, and membrane-associated functions of FAs, including their roles in phospholipid structure organization, energy metabolism, and cellular signaling [4]. The emergence of liposomal drug delivery systems led to investigations into FA and FA-containing lipids as formulation additives that enhance membrane interactions, intracellular uptake, and therapeutic delivery [5]. A key milestone represented the development of FA–drug conjugates, where covalent linkage of FAs to bioactive compounds increased lipophilicity, membrane permeability, plasma circulation time, and utilization of endogenous transport processes such as albumin binding and lymphatic uptake [6]. More recently, the field has tilted toward advanced FA-based nanoplatforms, encompassing liposomes, lipid nanoparticles, self-nanoemulsifying drug delivery systems, and self-assembling lipid-based prodrugs. This shift moves from the incidental use of FAs as mere structural excipients toward their rational design as pharmacokinetics and bioactivity modulators [7,8]. In oncology, this evolution is particularly important because FA-based strategies can simultaneously improve drug solubility, biodistribution, accumulation at the tumor site, and controlled release [9]. Moreover, in the case of specific polyunsaturated fatty acids (PUFAs), cancer-related processes such as oxidative stress, lipid metabolism, apoptosis, and ferroptosis can be directly influenced [10].

Hence, the historical trajectory of FA-based innovations highlights the integration of lipid science, medicinal chemistry, and nanotechnology to enable more selective and effective anticancer therapies.

Beyond their structural role in lipid metabolism, FAs possess physicochemical and biological properties that make them particularly attractive for oncological applications. Their amphiphilic structure, adjustable chain length and branches, unsaturation degree, and natural affinity for biological membranes and serum albumin provide FA with unique pharmacokinetic advantage in terms of drug absorption, distribution, and intracellular uptake [11,12]. These features can be exploited in drug design to improve the pharmacokinetics of anticancer drugs to enhance membrane permeability, prolong systemic circulation, redirect absorption pathways (including lymphatic transport), and modulate tissue distribution [13,14]. Improved pharmacokinetic performance, therapeutic efficacy and targeted release of various anticancer agents have been achieved through strategies such as: (i) covalent conjugation of FAs to therapeutic agents to generate lipophilic prodrugs and (ii) incorporation of FAs or FA-derived lipids into lipid-based nanocarriers (liposomes, LNPs, self-emulsifying nanosystems, and self-assembled lipid prodrugs known as LAPs) [15,16,17]. Moreover, in oncology research, specific FAs, and in particular polyunsaturated FAs (PUFAs), have been reported to exert intrinsic biological effects that can influence cancer cell viability, tumor cell proliferation, oxidative stress pathways, and cell death mechanisms [18]. Thus, in addition to their proved use in drug design as delivery aids for anticancer agents, FAs can also be viewed as a novel complementary approach in the treatment of various cancers [18,19].

The aim of this study is to review the available evidence on FA-based strategies in oncology, including FA–drug conjugation chemistry, lipid-based delivery systems, and to address the mechanistic rationale that supports the therapeutic potential of FA in cancer therapy. This review is structured in five distinct chapters that address: (1) background on FAs and their application in oncology, (2) the synthetic strategies underlying FA–drug derivatization and conjugation chemistry, (3) FA-based formulations, (4) the application of FA–drug conjugates in oncology, highlighting representative therapeutic examples, and (5) therapeutic application and biological effect of FA in cancer.

## 2. FA–Drug Conjugation: Chemical Strategies, Activation, and Transport

### 2.1. Chemistry Toolbox

FA conjugation represents one of the most widely used strategies in lipophilic prodrug design, enabling the modulation of physicochemical and pharmacokinetic properties. Specifically, through the covalent attachment of FAs to active drugs, parameters such as lipophilicity, membrane permeability, systemic circulation, metabolic stability, and tissue distribution can be systematically adjusted and improved [14]. Conjugation with FAs provides the resulting compound with a hydrophobic hydrocarbon domain that increases the drug’s overall lipophilicity. Key FA properties such as chain length, degree of unsaturation, and intrinsic albumin-binding tendency are able to directly influence membrane permeability, passive diffusion, and systemic distribution for the resulting conjugate. By modulating these structural features, FA–drug conjugates can benefit from endogenous transport pathways, exhibit prolonged blood circulation, and fine-tune tissue exposure. This ability to control absorption, distribution, metabolism, and excretion (ADME) parameters allows FA-based prodrugs to be specifically tailored for various objectives, including the improved uptake of poorly permeable drugs, sustained systemic exposure, and reduced pharmacokinetic variability [20].

Esterification represents the most straightforward route for designing FA–drug conjugates, being compatible with a broad spectrum of molecular scaffolds, as FAs can readily react with drugs containing hydroxyl or phenolic groups. Several synthetic strategies are available to generate FA–drug esters (Table 1), including the activation of the FA carboxyl group into more reactive intermediates such as acid chlorides or symmetric anhydrides, carbodiimide-mediated coupling, or alternative reactions, such as transesterification when compatible functional groups are present. These synthetic strategies facilitate the preparation of structurally diverse FA–drug esters and allow the precise modulation of their lipophilicity and interactions with biological membranes. The systematic variation of the fatty acid chain length or degree of unsaturation results in predictable alterations in its hydrophobicity and membrane affinity [21]. Such tunability is a defining advantage of FA-based prodrugs, as the physicochemical properties of the FA moiety can be deliberately selected to modulate absorption, passive diffusion, or tissue distribution [21].

The FA component should therefore not be regarded only as a hydrophobic appendage but also as a structural element that influences both the conjugation reaction and the biological performance of the final prodrug. Accordingly, the following sections discuss the main reaction mechanisms involved in FA–drug conjugation, as well as the effects of FA chain length and unsaturation on reactivity and physicochemical behavior.

#### 2.1.1. Activation and Esterification Using Acid Chlorides and Anhydrides

**Figure 2:**
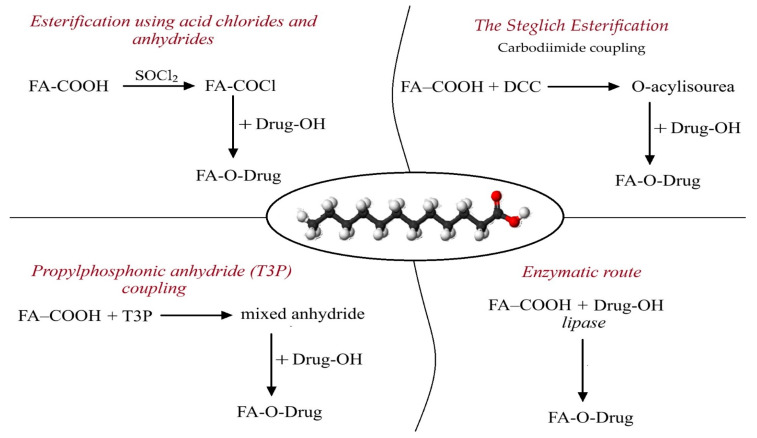
Synthetic strategies for FA–drug conjugation.

Converting FAs into their corresponding acid chlorides or symmetric anhydrides constitutes a long-standing and reliable approach for generating ester-type FA prodrugs (Figure 2). The synthesis of fatty acid chlorides involves the use of thionyl chloride or oxalyl chloride as reaction partners; the resulting highly reactive acyl chlorides are able to engage in efficient nucleophilic substitution with hydroxyl-containing drugs in order to form ester conjugates [27]. The approach provides a direct, high-yielding route to FA–drug esters; however, the strong electrophilicity of acyl chlorides can lead to side reactions when multifunctional drug molecules are involved (Table 1). In addition, esterification reduces free FA carboxylate, which may negatively influence albumin binding and, subsequently, pharmacokinetic behavior [28]. Although less reactive than acid chlorides, anhydrides provide operational advantages, including fewer side reactions involving acidic by-products [29]. Their moderate reactivity enables greater control when drug molecules contain additional functional groups; the activation strategies operate through well-understood acyl substitution mechanisms that are highly valued for their predictable behavior and efficiency [30]. From a mechanistic perspective, ester formation via acid chlorides and anhydrides proceeds through a classical nucleophilic acyl substitution pathway. The hydroxyl group of the drug acts as a nucleophile, attacking the electrophilic carbonyl carbon of the activated fatty acid derivative to form a tetrahedral intermediate, followed by elimination of chloride or carboxylate leaving groups, respectively. The high reactivity of acyl chlorides is attributed to the strong electron-withdrawing effect of the chlorine atom, which enhances the electrophilicity of the carbonyl carbon and facilitates rapid acyl transfer reactions [31].

#### 2.1.2. The Steglich Esterification

Carbodiimide-mediated esterification (the Steglich esterification) (Table 1) represents one of the most widely applied strategies for building ester links in FA–drug conjugates owing to its mild reaction conditions and compatibility with diverse functional groups [14]. In this method, activating agents such as dicyclohexylcarbodiimide (DCC) or 1-ethyl-3-(3-dimethylaminopropyl)carbodiimide (EDC) are used to convert the FA carboxyl group into a reactive O-acylisourea intermediate. In the presence of catalytic 4-dimethylaminopyridine (DMAP), the intermediate is transformed into a more electrophilic species, acyl-pyridinium, that facilitates an efficient nucleophilic attack on the drug’s hydroxyl group, leading to efficient ester bond formation (Figure 2). The reaction proceeds under relatively mild, near-neutral conditions, making it suitable for the derivatization of structurally complex or sensitive drug molecules. However, the efficiency of this reaction may be influenced by the steric accessibility of both the fatty acid carboxyl group and the drug hydroxyl group, particularly when long-chain or highly unsaturated fatty acids are used, as these structural features may affect solubility, conformational flexibility, and the approach of the nucleophile to the activated acyl intermediate [23,32].

#### 2.1.3. Propylphosphonic Anhydride (T3P)

T3P, a mild dehydrating agent, has emerged as an efficient coupling reagent for ester and amide bond formation involving FAs (Figure 2). T3P activates the FA carboxyl group by generating reactive mixed anhydrides that act as intermediates which enable nucleophilic attacks by alcohols or amines, under mild conditions, without the need for strong acids or elevated temperatures, thus providing a coupling environment that minimizes the racemization and/or the degradation of sensitive substrates [33]. T3P enables the formation of amide and ester bonds across a wide substrate spectrum, including sterically hindered or multifunctional amines, alcohols, and heterocyclic substrates [34]. T3P facilitates efficient coupling reactions even when classical carbodiimide reagents such as DCC fail or lead to complex mixtures; as an example, T3P enabled the clean conversion of diethylphosphonoacetic acid into phosphonacetamides, whereas DCC produced multiple side-products that reduced the overall reaction yield [34]. Furthermore, the reagent produces water-soluble by-products, thus simplifying work-up steps and allowing the efficient isolation of the desired products without chromatographic purification, a notable advantage for lipophilic intermediates typically difficult to purify [35]. The mild reaction conditions and reduced risk of rearrangement reactions make T3P particularly suitable for late-stage derivatization of sensitive drug molecules and for synthesizing structurally complex lipid prodrugs [33,34,35] (Table 1). T3P-mediated coupling proceeds through the formation of reactive mixed phosphonic anhydride intermediates, which increase the electrophilicity of the fatty acid carbonyl group and facilitate nucleophilic attack by alcohols or amines. The efficiency of this transformation is influenced by steric accessibility and substrate structure, as bulky or long-chain fatty acids may hinder the nucleophilic approach, while flexible or unsaturated chains can improve reactivity under mild conditions.

#### 2.1.4. Enzymatic Routes: Lipase-Catalyzed Esterification

Lipase-catalyzed esterification represents a selective and environmentally friendly alternative to chemical esterification, as lipases remain active across a broad range of conditions, including aqueous–organic interfaces and non-aqueous media (Figure 2). Their catalytic mechanism involves the formation of an acyl-enzyme intermediate, followed by the nucleophilic attack of an alcohol, enabling efficient coupling of FA with structurally diverse substrates while preserving sensitive functional groups [36]. The selectivity of lipases toward specific FA or alcohols is attributed to their preserved catalytic triad (Ser-His-Asp) and structural flexibility of the lid domain, which controls the access to the active site and contributes to high region and stereo selectivity during ester formation [37]. This structural adaptability allows lipases to esterify primary and secondary alcohols under conditions that would otherwise lead to degradation or side reactions with conventional chemical reagents [38,39]. When immobilized on hydrophobic supports, lipases demonstrate improved thermal stability, operational robustness, and reusability, features that are advantageous for continuous esterification or large-scale synthesis [18]. Immobilized systems also reduce diffusion limitations and allow higher substrate loading, which are critical parameters when esterifying long-chain FAs (LC-FAs) with limited solubility. A major advantage of enzymatic esterification is the minimal requirement for protective groups, as lipases display high chemoselectivity even in the presence of multiple reactive functionalities [37]. The efficiency of lipase-catalyzed esterification is also influenced by the structural features of the fatty acid substrate, including chain length and degree of unsaturation. Long-chain fatty acids may exhibit reduced diffusion and limited accessibility to the enzyme active site due to their hydrophobic nature, while unsaturated fatty acids, owing to their increased conformational flexibility, may better accommodate within the active site and enhance catalytic efficiency. These factors can significantly impact substrate recognition, binding, and overall reaction rates [39,40]. These features make lipase-mediated esterification attractive for synthesizing FA conjugates of structurally sensitive drugs or compounds containing multiple functional groups [36,41]; however, there are some disadvantages that must be taken into account, such as loss or inhibition of enzyme activity, which is a complex and costly downstream processing that involves the separation of byproducts and slow reaction times [42,43] (Table 1).

Overall, FA structure is a major determinant of biological performance. Longer-chain FAs generally increase lipophilicity, albumin affinity, and circulation time but may reduce aqueous solubility and slow enzymatic access. Unsaturated FAs increase conformational flexibility and membrane interactions, while polyunsaturated FAs may additionally influence oxidative stress and lipid-peroxidation-related cell death. Branching and linker position can further modify steric accessibility, self-assembly, albumin binding, and enzymatic release. Therefore, FA structure should be considered a design variable that controls pharmacokinetics, membrane affinity, and biological activity.

### 2.2. Alternative Linkers Beyond Classical Esters

**Figure 3:**
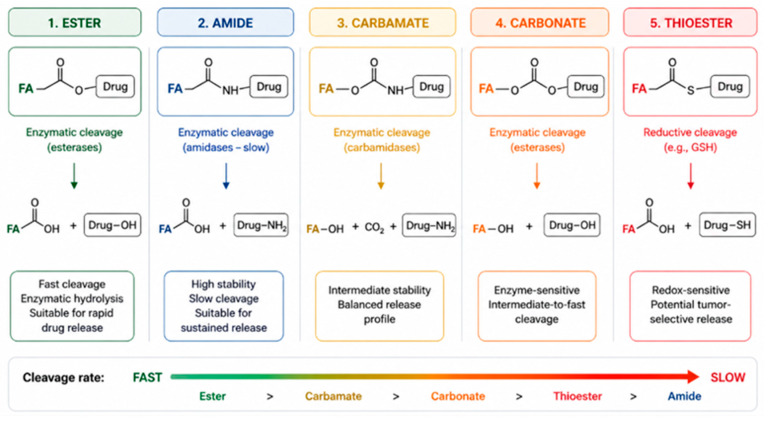
Representative linker strategies used in FA–drug conjugates and their impact on drug release.

In FA–based prodrug design, alternative linkages beyond classical esters, such as amides, carbamates, carbonates, and thioesters (Figure 3), offer opportunities to modulate metabolic stability, control drug release, and adjust pharmacokinetic behavior in a predictable manner. The general principles of prodrug optimization emphasize that the chemical nature of the linker is one of the most decisive determinants of the activation rate, cleavage pathway, and tissue exposure [44].

FA–based derivatization can also proceed through amide bond formation, in which the carboxyl group of the FA is coupled to an amino functionality on the drug molecule. In comparison with esters, these amide conjugates display substantially higher resistance to hydrolysis, reflecting their intrinsically higher chemical and enzymatic stability. Amide linkers are mainly cleaved by amidase- or peptidase-like enzymes and are therefore usually activated more slowly than esters [45]. Consequently, FA amide conjugates exhibit prolonged systemic circulation but slower or incomplete enzymatic activation. This feature is highlighted in analyses of FA–linked prodrugs, where amide derivatives show prolonged persistence and, in many cases, minimal in vivo hydrolysis due to the limited activity of endogenous amidases toward lipid conjugates [46,47]. This reduced susceptibility to hydrolysis can limit their usefulness in applications requiring efficient or predictable prodrug activation. However, the enhanced stability of FA amides may be beneficial when sustained exposure, increased albumin interaction, or altered tissue distribution are desired [48,49]. In these cases, the amidic moiety can function less as a classical cleavable prodrug linker and more as a structural modification designed to achieve prolonged pharmacokinetic effects [50]. While this increased stability can be advantageous for maintaining sustained systemic levels or enhancing albumin association, it restricts their applicability in strategies requiring rapid or controlled enzymatic activation [49].

Carbamate linkers provide an intermediate stability profile between esters and amides. Their cleavage is generally mediated by esterases or related hydrolases, producing gradual release of the parent drug. Thus, they are less prone to spontaneous hydrolysis than esters yet remain susceptible to enzymatic cleavage, enabling more predictable release kinetics [51]. Prodrug frameworks describe carbamates as versatile linkers suitable for improving oral absorption, enhancing chemical stability, and reducing premature hydrolysis, especially in the case of polar or nucleoside-based anticancer drugs [52]. In lipid prodrug systems, carbamates have been shown to prolong circulation time while still enabling the intracellular delivery of the active molecule through enzymatic pathways, thus offering a balance between stability and drug release [53].

Carbonate linkers share mechanistic similarities with carbamates but generally undergo cleavage more readily. Carbonates are also hydrolyzed by esterases or related hydrolases, often faster than carbamates but slower or more controlled than simple esters. Their susceptibility to enzymatic hydrolysis makes them suitable for applications requiring moderate stability in plasma and efficient release in tissues. Reviews of lipid-based conjugates indicate that carbonate-modified prodrugs can improve membrane permeation and passive uptake, which is particularly relevant for hydrophilic anticancer agents that require enhanced cellular entry [49,54,55]. Carbonate linkers offer synthetic flexibility for attaching FA in different positions without altering the essential functionality of the parent drug.

Thioesters, although less frequently used, provide unique advantages as releasable handles because they undergo rapid cleavage in reductive intracellular environments. Thioesters can be cleaved by thioesterases or by intracellular thiols such as glutathione, supporting release in reductive tumor-cell environments. Prodrug design principles emphasize that thioester stability in plasma contrasts with their enhanced susceptibility to cleavage by intracellular thiols such as glutathione, enabling site-selective activation in tumor cells where reductive stress is elevated [44,56].

In the context of FA conjugation, thioesters support selective intracellular release while benefiting from lipid-driven passive uptake and albumin-mediated transport. They have been used to attach lipids to antitumor antibiotics and nucleoside analogues as part of strategies to enhance tumor accumulation and minimize efflux transporter recognition [55]. An overview of representative FA derivatization and linker-based conjugation strategies is presented in Figure 3 and summarized in Table 2.

### 2.3. Enzymatic Activation and Prodrug Release

The following subsections focus on the activation and transport of FA–drug conjugates. These processes depend on linker type and FA structure and describe how these properties affect enzymatic release, albumin binding, biodistribution, and lipid-mediated absorption.

**Figure 4:**
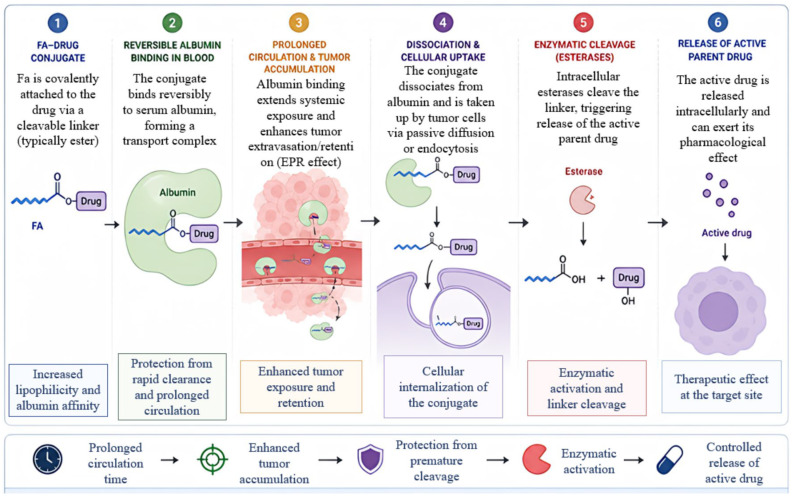
Albumin-mediated transport and enzymatic activation of FA–drug conjugates.

A common rationale for FA–modified prodrugs and conjugates is to exploit their reversible binding to serum albumin, which acts as an endogenous carrier, thereby increasing the apparent molecular size, reducing renal clearance, and improving systemic exposure. Albumin binding can prolong circulation and increase tumor exposure [59]. At the mechanistic level, albumin binding relies on dynamic and reversible interactions that allow FA-modified conjugates to circulate in the bloodstream predominantly in an albumin-bound state while retaining the capacity to dissociate and distribute into tissues. Albumin affinity depends on FA structure and linker design [59,60,61]. Albumin association can further modulate these processes by partially shielding FA–drug conjugates from enzymatic access in circulation, thereby reducing premature cleavage before tissue distribution [62]. Even conjugates with comparable albumin-binding affinities may display markedly different stability and release profiles depending on the attachment site and linker chemistry, thus highlighting the role of molecular design in shaping the drug’s pharmacokinetic behavior and activation timing [60,61]. The overall mechanism of albumin-mediated transport, tissue distribution, enzymatic activation, and active drug release from FA–drug conjugates is schematically illustrated in Figure 4.

In parallel, drug release from FA-modified systems is most often mediated by enzymatic cleavage, typically through ester hydrolysis catalyzed by ubiquitous plasma and tissue esterases [63]. The efficiency of enzymatic processing is strongly dependent on the site and chemistry of FA attachment. Conjugation via phenolic hydroxyl groups generally yields more labile esters with faster cleavage rates, whereas attachment through primary aliphatic hydroxyl groups confers intermediate stability [64]. The rate of enzymatic activation strongly depends on linker chemistry, with ester bonds generally cleaving faster than more stable amide or carbamate linkages [45].

An essential feature of FA–drug ester conjugates is the controlled release of the parent drug following enzymatic cleavage. The significance of enzymatic hydrolysis in the activation of FA–drug esters is supported by evidence that shows that carboxylic esters are highly susceptible to undergo biotransformation by multiple classes of human hydrolases, such as carboxylesterases, cholinesterases, paraoxonases, and related esterases located in plasma, liver, intestine, and other tissues, able to facilitate the rapid and predictable cleavage of ester bonds, thus enabling the controlled release of the parent drug [65]. The hydrolysis rate can be modulated by several structural parameters, including the steric environment around the ester bond, the nature of the FA chain and the leaving-group properties of the drug moiety. This generally predictable enzymatic cleavage behavior allows FA–ester prodrugs to be tailored for rapid, moderate, or sustained release profiles, respectively, depending on specific therapeutic requirements [49].

### 2.4. Lymphatic Transport and Lipid Absorption

Beyond influencing drug release, FA conjugation can also modify the pathways in drug absorption. FA–drug conjugation is highly relevant within lipid-mediated drug delivery frameworks, as increasing molecular lipophilicity through FA attachment can redirect the drug absorption pathways toward the intestinal lymphatic system rather than the portal circulation [66]. Such a shift allows the conjugated drug to bypass the first-pass hepatic metabolism. This phenomenon is attributed to the preferential association of LC lipids with lipoprotein assemblies in enterocytes [67]. LC-FA conjugates tend to be incorporated into chylomicrons, thereby promoting lymphatic transport, improving systemic bioavailability, and influencing tissue distribution profiles. Experimental studies show that lipidic prodrugs bearing optimized FA moieties are efficiently packaged into chylomicrons produced by enterocyte-like cells. These prodrugs exhibit increased lymphatic uptake and enhanced exposure in extrahepatic tissues such as the lungs, thus demonstrating the impact of FA-mediated lipophilicity on systemic distribution patterns [68]. Overall, FA conjugation modifies drug delivery and can also redirect drug absorption by promoting drug association with lipid transport mechanisms such as chylomicron formation and lymphatic transportation. Consequently, FA derivatization represents a versatile strategy not only to increase drug lipophilicity but also to modulate absorption pathways, systemic half-life, tissue targeting, and release kinetics.

When taken as a whole, these derivatization techniques show that FA conjugation provides a flexible chemical platform for adjusting the pharmacokinetics, activation kinetics, and physicochemical characteristics of drugs. Beyond direct FA–drug conjugates, FAs also play an important role as structural components of lipid-based nanocarriers designed to improve anticancer drug delivery.

The next section discusses these formulation strategies.

## 3. FA-Based Formulations for Anticancer Delivery

### 3.1. Liposomal Systems Containing FA

Liposomal formulations are much more effective in delivering FA into cells compared to free FAs [69]. To observe the efficacy of liposomes as delivery vehicles, Hosick’s et al. compared three oleic acid (OA) formulations; the results obtained indicated that liposomes, composed of phosphatidylcholine (PC), phosphatidylserine (PS) and OA (10:1:10 molar ratio), increased the OA absorption rate into cells compared to its free form or albumin-complexed oleic acid, becoming a basis for the development of FA formulations for the administration of anticancer drugs [70]. Later, this liposomal system was formulated as a liposomal hydrogel doped with PUFA, decosahexaenoic acid: Lp(DHA)@CP Gel, loaded with photosensitizer (Ce6) and anti-PD-L1 antibody and used for local phototherapy immunotherapy in the treatment of a 4T1-luc breast cancer model in BALB/c mice [71]. The liposomal hydrogel group exposed to laser irradiation demonstrated an almost complete ablation of the tumor; additionally, the liposomes exhibited both stability and proper cell interaction, a uniform particle distribution, which led to a homogeneous compound loading and constant release.

A recent study that used quercetin (QUE) and folic acid formulated with eicosapentaenoic acid (EPA)-based liposomes with PC and choLNOlesterol (col) (7:4:1 molar ratio) was tested in vitro in healthy HEK293 cells as well as in HeLa and HepG2 cancer cells [72]. The formulation showed low toxicity toward healthy cells and strong cytotoxicity against HeLa and HepG2 cancer cells, with IC_50_ values in the micromolar range; compared with free QUE, the liposomal system exhibited higher cytotoxic effects. The authors suggest that this effect can be attributed to increased encapsulation efficiency and reduced particle size. Moreover, the combination of acidic pH and ROS destabilized the liposomes and triggered lipid peroxidation that destroyed the integrity of the liposomal membrane, allowing the drug to penetrate the target site in a controlled manner [72]. Kim et al. designed and tested a structurally reinforced liposomal system consisting of a LC saturated phospholipid (DSPC) and 1,2-distearoyl-sn-glycero-3-phosphoethanolamine (DSPE), used in conjugated form with polyethylene glycol (DSPE-PEG2000), cholesterol, and DHA-PC, which optimized membrane rigidity, circulation time, and release efficiency [73]. The NPs (100–120 nm, slightly negative zeta potential) achieved ~95% encapsulation of doxorubicin (DOX) and irinotecan; the in vitro studies in CT26 colon cancer cells revealed superior cytotoxicity compared to free DOX, while in vivo experiments in BALB/c mice demonstrated a fourfold reduction in tumor volume compared to the free drug [73]. Of note, tumor drug accumulation was doubled (12 μg/g vs. 6 μg/g), indicating that FA incorporation enhances tumor-specific delivery rather than merely increasing systemic exposure. Similarly, Kumar et al. developed an alpha-linoleic acid (ALA)-PEGylated liposomal system for paclitaxel (PTX) delivery; the formulation exhibited favorable physicochemical properties, allowing high colloidal stability and 88.6% encapsulation efficiency [74]. The in vitro tests performed in MCF-7 breast cancer cells revealed a significantly lower IC_50_ value (4.1 μg/mL) for the liposomal system compared to the free PTX, indicating the superior anticancer activity of the liposomal formulation [74]. In vivo, the reduction in tumor volume was substantially greater for the liposomal formulation vs. free PTX, while minimal albumin interaction provided prolonged circulation [74]. Dupertuis et al. used PUFA-based liposomes containing DHA and EPA (1:2 molar ratio) to improve the intracellular delivery of 5-Fluorouracil (5-FU) [75]. The liposomal system exhibited high stability in solution, prevented the formation of aggregates and significantly enhanced cytotoxicity in LS174T and HT-29 colorectal cancer cells compared to free 5-FU [75]. Using a distinct strategy, Brody et al. developed PEGylated cationic LNPs (LITA-CAN) that incorporated 1,2-dioleoyl-3-trimethylammonium-propane and OA derivatives [76]. The system had a positive zeta potential which allowed the active substance to be released before lysosomal degradation. In HCT116 colon tumor cells, the system induced a 50% decrease in cell viability trough apoptosis, while in murine xenograft models, the treatment reduced tumor volume by approximately 50% compared to control [76].

As summarized in Table 3, FA-containing liposomes generally showed suitable nanoscale size, acceptable colloidal stability, and high encapsulation efficiency. Their biological performance was mainly associated with improved cellular uptake, controlled release, and increased tumor accumulation.

### 3.2. LNPs

Zhu et al. reported the development of a hybrid LDL-based NPs containing FAs (linoleic and oleic acid) to optimize DOX delivery [77]. The small particle size (18–25 nm) enabled LDL receptor-mediated uptake in A549 lung cancer cells, resulting in a sixfold lower IC_50_ value compared to the free DOX. In parallel, the healthy NIH-3T3 cells were minimally affected, thus revealing a selective anticancer activity for FA-tailored LNPs. The in vivo experiments with fluorescence tracking revealed tumor accumulation as well as prolonged circulation [77]. FA-derived LNPs are not limited to the transportation of cytotoxic drugs but can also facilitate advanced immunotherapeutic strategies. As described by Li et al., ionizable FA-LNPs composed of SM-102, DSPC, cholesterol and DMG-PEG 2000 and functionalized with anti-CD3 antibodies were able to deliver mRNA encoding tumor-recognizing receptors and enable the immune-mediated cell death of B16-F10 murine melanoma cells in a selective manner; in vivo, the FA-LNP system significantly reduced tumor size [78].

Compared to conventional liposomes, FA-LNPs exhibit smaller or comparable sizes (~26–140 nm) with narrow PDI values (0.12–0.17), indicating highly homogeneous nanostructures (Table 4). The LDL-based liposomes (~26 nm) can be particularly considered for their receptor-targeting capability. In LNPs, although drug loading varies (~2–25%), the encapsulation efficiency approaches ~100%, thus emphasizing the structural difference between LNPs and liposomes: LNPs rely on lipid core entrapment and ionizable lipids rather than the liposomal aqueous core encapsulation. Zeta potential values vary around neutrality (−15 to +2 mV), reducing nonspecific plasma interactions and facilitating extended systemic circulation (up to 2–4 days in some systems). PEGylation further minimizes the immune clearance and protein adsorption (Table 4). Moreover, PEGylated ionizable LNPs display near-neutral surface charge, pH-responsive release, and preferential splenic accumulation associated with immune-cell targeting; FA-core LNPs enable intracellular drug activation through LDL receptor-mediated endocytosis followed by metabolization of the FA core, thus resulting in the rapid and controlled release of the payload while protecting it against lysosomal degradation.

### 3.3. Self-Nanoemulsifying Drug Delivery Systems (SNEDDS)

In addition to the liposomal DSPC/DOPE/cholesterol ± FA system and FA-LNPs, researchers also developed SNEDDS that use a mixture of oils as solvents and FAs as surfactants with the goal of transferring insoluble drugs into the blood. Chaudhuri et al. [79] formulated an omega-3 oil-based SNEDDS to improve the oral delivery of docetaxel. The nanoemulsion (~120 nm, negative zeta potential) doubled drug release across dialysis membranes and enhanced its intestinal absorption in goat duodenum models [79]. In order to increase the systemic exposure of a poorly bioavailable drug, Bravo-Alfaro et al. incorporated betulinic acid in lauroglycol FCC, a nonionic water-insoluble surfactant (SNEDDS-L), as well as in caprylic acid, a saturated medium-chain FA (SNEDDS-C) [80]. Both systems showed highly uniform and stable NPs. In vitro tests performed using enzymes that mimic the gastrointestinal environment demonstrated that both SNEDDS were stable at acidic pH with particle size remaining constant, while in vivo tests revealed a 15-fold increase in the bioavailability of the BA incorporated in SNEDDS compared with free BA; overall, the experiments supported the use of SNEDDS [80]. Similarly, medium-chain (MCT) and LC triglyceride (LC-T) SNEDDSs were compared for PTX delivery [81]. The authors reported a smaller particle size for the LCT-based formulation (75.4 nm), compared to the MCT-based formulation (105.6 nm), but a uniform particle distribution and a positive zeta potential that facilitated mucoadhesion and enhanced absorption for both systems [81]. Both formulations significantly increased intestinal permeability and plasma drug levels, with LCT systems showing slightly superior performance in ex vivo conditions on small intestine isolated from Wistar rats [81]. These findings suggest that FA chain length and oil composition critically influence the efficiency of oral delivery.

SNEDDS formulations exhibited particle sizes between ~22 and 140 nm with low PDI values, thus confirming stable nanoemulsion formation (Table 5). Unlike liposomal systems, the encapsulation efficiencies typically reached ~100% due to the complete drug solubilization in the oil phase. However, the drug loading values were generally lower compared to self-assembling prodrugs. Zeta potential values ranged from moderately negative (−12 to −18 mV) to positive (+18 to +22 mV), depending on the surfactant composition. Positively charged systems revealed an enhanced mucoadhesion and intestinal permeability, which explains the significantly increased plasma drug levels reported in ex vivo and in vivo studies. Moreover, enzyme-triggered release was rarely observed in SNEDDS systems; drug release occurred through passive solubilization and diffusion. Nevertheless, advanced formulations introduce pH- and redox-responsive mechanisms, suggesting ongoing evolution toward more controlled delivery strategies.

### 3.4. Self-Assembling LAPs

Gupta et al. [82] developed a stearic acid-modified prodrug NPs that self-assembled without the need for conventional carriers. This system, containing a lipophilic FA derivative of gemcitabine (4-(N)-stearoyl gemcitabine—C18dFdC), was incorporated into poly-lactic-co-glycolic acid NPs (PLGA NPs). In vitro and in vivo tests revealed that, compared to the parent drug, the FA prodrug improved uniformity, electrostatic stability and the controlled release of the active substance, as well as increased biological half-life and cytotoxic effects against the MCF-7 breast cancer cell line, while reducing systemic toxicity [82]. In a similar manner, Zhong et al. [83] reported on a PTX-linoleic acid (CLA-PTX) system capable of self-assembly into stable LAPs (~105 nm, negative zeta potential) without requiring a transporter. CLA-PTX LAP proved to be safer than the commercial formulation Taxol, with reduced systemic toxicity and enhanced cytotoxicity compared to free PTX in B16-F10 melanoma, MDA-MB-231 breast and U87-MG glioma cancer cells [83]. In order to test if the anticancer activity increases with FA chain length, Ngo et al. [84] developed LAPs of leuprolide with different chain length FAs: lauric acid (C12), palmitic acid (C16) and stearic acid (C18). The results confirmed that increasing the FA chain length from C12 to C18 improves the anticancer efficacy and stability of leuprolide compared to the free drug, with stearic acid conjugates achieving nearly complete tumor inhibition [44]. In support of such results, Zhang et al. [85] formulated LAPs of PTX with branched FAs (BFAs—BFA C16, BFA C18 and BFA C20) and indicated that branched FA conjugation enables redox-responsive release and prolonged circulation, while minimizing systemic toxicity. The LAPs with branched FA outperformed free PTX in terms of cytotoxic activity in 4T1 murine breast cancer cells, with BFA C18 having superior antiproliferative effects compared to the other FA [85].

LAPs exhibit particle sizes ranging from ~68 to ~318 nm, with PDI values typically below 0.2, thus indicating controlled self-assembly driven by FA conjugation. A defining characteristic (Table 6) is their exceptionally high encapsulation efficiency (~98–100%) and significantly higher drug loading (often 40–80%) compared to liposomal systems. The zeta potential predominantly fell within −22 to −45 mV, reflecting strong electrostatic stabilization. Interestingly, increasing the FA chain length (C12 → C18 → branched FA systems) correlates with improved tumor accumulation and prolonged circulation, as reflected in the biodistribution data. The release mechanisms are predominantly enzyme-, redox-, or hydrolysis-triggered; this controlled intracellular activation differentiates LAPs from conventional lipid carriers. Covalent FA–drug linkage prevents premature leakages and allows selective cleavage within TME (e.g., via GSH, ROS, or lysosomal enzymes). Albumin interaction further supports LAP stability and tumor exposure.

**Figure 5:**
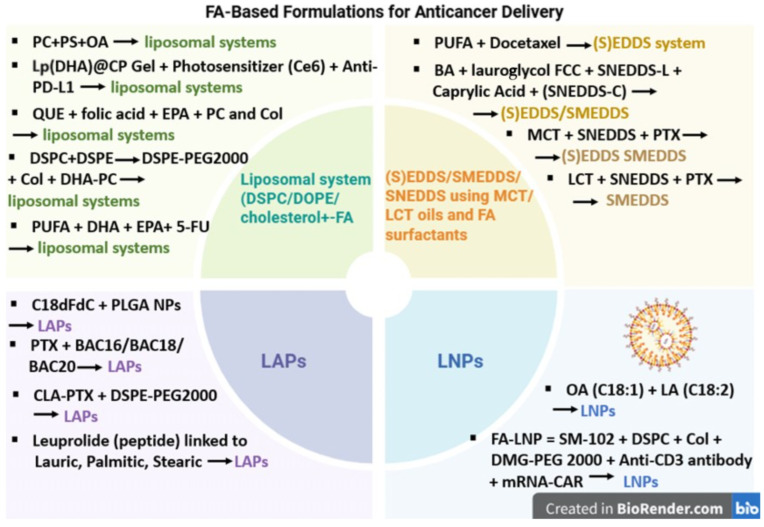
Overview of FA-based lipid nanocarrier systems used for anticancer drug delivery. 5-FU: 5-Fluorouracil; Anti-PD-L1: Anti-Programmed Death-Ligand 1 Antibody; BA: betulinic acid; BAC: Branched Alkyl Chain; C18dFdC: 4-(N)-stearoyl gemcitabine; Ce6: Chlorin e6; CLA: Conjugated linoleic acid; Col: Cholesterol; DHA: Docosahexaenoic acid; DMG: Dimyristoyl Glycerol; DSPC: Distearoylphosphatidylcholine; DSPE: Distearoylphosphatidylethanolamine; EPA: Eicosapentaenoic acid; FA-LNP: Fatty Acid–Lipid Nanoparticles; FCC: Food Chemicals Codex; LA: Linoleic acid; LAPs: Lipidic prodrugs; LCTs: Long-chain triglycerides; LNPs: Lipid nanoparticles; Lp(DHA)@CP: Liposomes loaded with Dihydroartemisinin Encapsulated; MCTs: Medium-chain triglycerides; mRNA-CAR: Messenger RNA encoding Chimeric Antigen Receptor; NPs: Lipid nanoparticles; OA: Oleic acid; PC: Phosphatidylcholine; PEG: Polyethylene glycol; PLGA: Poly(lactic-co-glycolic acid); PS: Phosphatidylserine; PTX: Paclitaxel; PUFA: Polyunsaturated fatty acid; QUE: Quercetin; SM-102: 1-octylnonyl 8-[(2-hydroxyethyl)[6-oxo-6-(undecyloxy)hexyl]amino]-octanoate; SNEDDS: Self-nanoemulsifying drug delivery systems; SNEDDS-C: Self-nanoemulsifying drug delivery systems and Capryol; SNEDDS-L: Self-nanoemulsifying drug delivery systems and Lauroglycol. Created in BioRender. Bătrîna, O. (2026) https://BioRender.com/tcvzlsy.

As summarized in Table 3, Table 4, Table 5 and Table 6, FA-modified nanocarriers generally showed suitable nanoscale size, acceptable colloidal stability, high encapsulation efficiency, and improved tumor-related delivery parameters. LAPs showed the highest drug loading, whereas liposomes and LNPs offered broader formulation flexibility. An overview of the representative FA–modified lipid nanocarriers for anticancer drug delivery is shown in Figure 5.

Overall, each FA-based delivery platform presents specific strengths and limitations. Liposomes are versatile and suitable for combination therapies, but their drug loading is generally lower, and release may depend strongly on bilayer composition. LNPs offer high encapsulation efficiency, controlled intracellular delivery, and suitability for nucleic acid or immunotherapeutic payloads, although their performance depends on ionizable lipid composition and PEGylation. SNEDDS are particularly useful for improving oral delivery, solubility, intestinal absorption, and lymphatic transport of hydrophobic drugs, but they provide less tumor-selective targeting than other nanosystems. LAPs provide high drug loading, carrier-free self-assembly, prolonged circulation, and stimulus-responsive release, but their applicability depends on successful covalent drug–FA conjugation and predictable intracellular cleavage. Therefore, liposomes and LNPs are most applicable for parenteral delivery and combination strategies, SNEDDS for oral delivery of poorly soluble drugs, and LAPs for prodrug-based approaches requiring high payload and controlled activation.

## 4. FA–Drug Conjugates in Oncology

While the previous section focused on lipid-based nanocarriers incorporating FAs, FAs can also be directly conjugated to anticancer drugs in order to generate lipophilic prodrugs with improved pharmacokinetic properties. Several FA–drug conjugates have been explored to enhance the therapeutic performance of the active drug. The following examples illustrate how FA conjugation has been applied across different classes of anticancer compounds.

### 4.1. DHA–PTX

Docosahexaenoic acid (DHA)–PTX (Taxoprexin) is a representative example of a FA–taxane conjugate developed to improve the therapeutic profile of PTX through albumin-mediated delivery and controlled enzymatic release. PTX is known to exhibit extensive plasma protein binding, particularly to serum albumin, which contributes to its wide systemic distribution but does not, on its own, overcome limitations related to solubility, toxicity, or suboptimal tumor accumulation. Building on this property, the conjugation of PTX with LC-FAs such as DHA has been explored as a prodrug design strategy in order to deliberately enhance albumin binding and, consequently, prolong circulation time [57]. In Taxoprexin, the DHA moiety serves as a lipid anchor that promotes its reversible binding to serum albumin, thereby increasing its apparent molecular size and reducing its rapid renal clearance. This prolonged circulation facilitates a greater accumulation of the conjugate in the tumor tissue, while the PTX payload remains masked during the systemic transport. The release of the active drug is achieved through the enzymatic cleavage of the ester link connecting DHA to the taxane core, thus enabling the regeneration of the free PTX at the active site [58]. The biological relevance of incorporating DHA as the FA component in drug conjugates is further supported by evidence that shows that LC-PUFAs can modulate cancer cell viability and apoptotic signaling pathways. DHA-based derivatives have been shown to reduce cancer cell survival and induce apoptosis in tumor models while maintaining low toxicity toward normal cells. For the DHA–PTX conjugate, this dual contribution of albumin-mediated delivery combined with favorable biological properties due to the FA moiety provides a mechanistic rationale for its improved antitumor activity compared to the parent drug [57].

### 4.2. FA–Nucleoside Conjugates: DHA–Gemcitabine

FA–nucleoside conjugates have been explored as a strategy to address key pharmacological limitations of nucleoside analogs such as gemcitabine, including its rapid systemic clearance, metabolic deactivation, and limited tumor selectivity. Gemcitabine remains a cornerstone chemotherapeutic agent in pancreatic cancer; however, its clinical efficacy is frequently compromised by the rapid emergence of drug resistance and dose-limiting toxicity. Conjugation of gemcitabine to LC-FAs, such as DHA, is designed to alter its biodistribution by increasing its lipophilicity and facilitating its association with endogenous lipid carriers, including serum albumin. PUFAs, including DHA, exhibit distinct biological activities that are relevant in the context of nucleoside-based chemotherapy. Experimental evidence shows that exogenous PUFAs can be efficiently incorporated into cancer cell membranes, where they modulate lipid metabolism and oxidative stress pathways. This lipid remodeling renders tumor cells more susceptible to oxidative damage and regulated cell death pathways, particularly ferroptosis, which is driven by lipid peroxidation. PUFA exposure has been shown to partially overcome gemcitabine resistance in pancreatic cancer models. In gemcitabine-resistant pancreatic cancer cell lines, the treatment with specific PUFAs induced delayed but measurable cytotoxic effects that were mechanistically linked to ferroptosis rather than apoptosis. These findings provide a mechanistic rationale for the design of FA–gemcitabine conjugates, where the FA moiety may not only improve drug pharmacokinetics and tumor exposure but also contribute biologically to the anticancer effect by priming tumor cells toward lipid-peroxidation-driven death pathways. Differences in PUFA structure were shown to influence the magnitude and kinetics of the drug’s antitumor activity. While DHA exhibited a comparatively milder ferroptotic effect than arachidonic acid (AA) or EPA, its favorable safety profile and compatibility with long-term systemic exposure supports its selection as a conjugation partner for nucleoside analogs. Within FA–nucleoside conjugates, DHA may therefore serve a dual role, acting both as a delivery-enhancing lipid anchor and as a modulator of the tumor lipid metabolism [86].

### 4.3. FA Conjugates of Natural Products: PUFA–Resveratrol

Natural products with anticancer activity, such as resveratrol, have attracted sustained interest in the field of cancer therapy due to their pleiotropic biological effects and favorable safety profiles; however, their clinical translation has been limited by their poor aqueous solubility, fast metabolism, and low systemic bioavailability. Resveratrol exhibits its anticancer activity across multiple tumor types by modulating key signaling pathways involved in proliferation, apoptosis, inflammation, angiogenesis, and oxidative stress. Despite this broad biological activity, its rapid clearance and extensive first-pass metabolism substantially reduce effective tumor exposure following systemic administration. Conjugation of resveratrol to LC-PUFAs has been proposed as a strategy to overcome these pharmacokinetic limitations by increasing drug lipophilicity and promoting its association with endogenous lipid carriers. The lipid-based modification can enhance both the membrane affinity and the cellular uptake of polyphenolic compounds, thereby improving their intracellular availability at the tumor site. PUFA tagging is expected to facilitate the prolonged systemic circulation and preferential accumulation of the active drug in lipid-rich tumor environments. At the cellular level, resveratrol influences the redox homeostasis as well as mitochondrial function, thus contributing to growth arrest and programmed cell death in cancer cells. Studies emphasizes that resveratrol can sensitize tumor cells to oxidative stress while sparing normal tissues, an effect that is particularly relevant when combined with lipid-driven delivery strategies. PUFA conjugation may further amplify these effects by integrating resveratrol into the lipid metabolic pathways that are frequently dysregulated in cancer [58]. Representative FA–drug and FA-assisted anticancer systems discussed in this section are summarized in Table 7.

## 5. Biological and Therapeutic Roles of FA in Cancer

FAs are increasingly recognized not only as structural components of lipid-based drug delivery systems but also as biologically active molecules capable of altering cancer development and the therapeutic outcome. Cancer cells display profound lipid metabolic reprogramming, including increased lipid uptake, de novo FA synthesis, altered cholesterol homeostasis, and lipid droplet accumulation, which supports cell proliferation and is mechanistically linked to drug resistance and impaired therapeutic response [57]. Thus, FAs can strengthen cancer therapy through multiple mechanisms, including the modulation of inflammatory signaling, the remodeling of membrane lipid composition, the induction of oxidative stress pathways, and the sensitization of tumor cells to anticancer treatments. Understanding these biological roles is essential in order to correctly interpret the therapeutic strategies discussed in the previous chapters, where FAs are employed as prodrug components, lipid carriers, or structural elements in nanoparticulated platforms.

### 5.1. Lipid Metabolism in Cancer

One of the hallmarks in cancer metabolism is the reprogramming of the lipid metabolic pathways that enable cancer proliferation, tumor growth, survival and metastasis [87]. Rapidly proliferating tumor cells require large quantities of lipids able to sustain membrane biosynthesis, energy production, and signaling processes. Consequently, cancer cells frequently exhibit increased uptake of exogenous fatty acids, enhanced de novo lipogenesis, and accumulation of lipid droplets [88]. These metabolic adaptations allow tumor cells to maintain a high proliferative rate and to withstand metabolic stress within the TME.

Beyond their structural role, the products of dysregulated lipid metabolism also have diverse pro-tumorigenic functions. For example, cholesterol intervenes in the formation and stability of specialized microdomains in the plasma membrane, called lipid rafts, that concentrate and facilitate the oncogenic signaling [89]. Others, like phosphatidylinositol phosphates (PIPs) serve as secondary messengers in the PI3K pathway, while AA derivatives promote inflammation, immune evasion and angiogenesis [90,91]. Once considered just deposit compounds, lipid droplets are now recognized as dynamic organelles that can protect against lipotoxicity and oxidative stress and can also provide a rapid energy source, especially in the high-energy demand context of cancer cells [92].

The high metabolic demand of cancer cells will proceed beyond de novo lipogenesis to include an enhanced uptake and oxidation of exogenous FA. Studies have shown that cancer cells upregulate CD36, a FA transporter, in order to scavenge lipids from the TME [93]. The newly incorporated FAs are either stored or used in the mitochondrial β-oxidation to produce ATP and NADPH [94].

**Figure 6:**
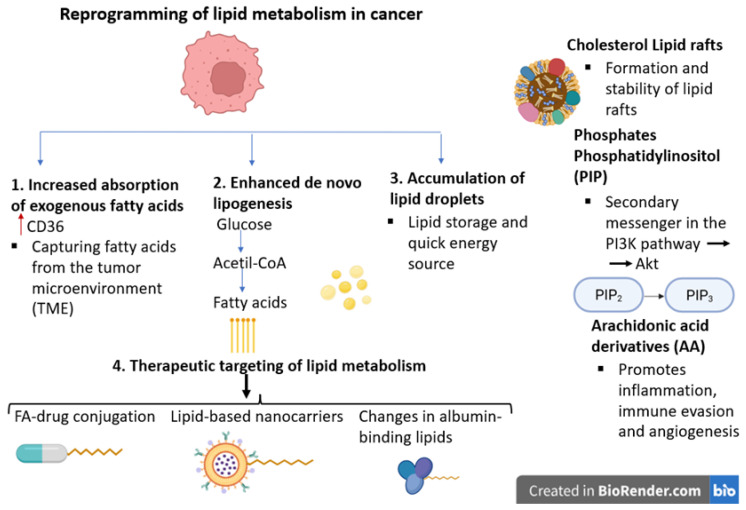
Reprogramming of lipid metabolism in cancer cells and therapeutic implications. AA: Arachidonic acid derivatives; Akt: Protein kinase B; CD3: Cluster of Differentiation 3; Phosphoinositide 3-kinase; PIP: Phosphates Phosphatidylinositol; TME: Tumor Microenvironment (Created in BioRender. Bătrîna, O. (2026) https://BioRender.com/5b33k1v).

Due to this lipid dependency, FA-related metabolic pathways have emerged as potential targets in cancer therapy. The strategies that exploit tumor lipid metabolism include FA–drug conjugation, lipid-based nanocarriers, and albumin-binding lipid modifications designed to alter drug biodistribution and tumor uptake. Such approaches leverage the intrinsic metabolic demands of cancer cells while simultaneously improving the pharmacokinetic properties of anticancer agents (Figure 6).

### 5.2. Antitumor Mechanisms and Effects of FAs

#### 5.2.1. Modulation of Lipid Metabolism in Cancer Cells

The occurrence of the metabolic rewiring observed in malign tumors is frequently driven by oncogenic signaling pathways that increase lipid synthesis, where key transcription factors such as the Sterol Regulatory Element-Binding Proteins (SREBPs) act as the main regulators of the lipid homeostasis. SREBPs are frequently hyperactivated in tumors through oncogenic signaling pathways such as PI3K/AKT/mTOR, leading to the coordinated overexpression of enzymes central to fatty acid and cholesterol synthesis [95,96]. This anabolic shift is driven by enzymes, such as ATP-citrate lyase (ACLY)—that catalyzes the conversion of the mitochondrial derived citrate into cytosolic acetyl-CoA, Acetyl-CoA Carboxylase (ACC)—that carboxylates acetyl-CoA to form malonyl-CoA, and Fatty Acid Synthase (FASN)—that adds two carbons from malonyl-CoA to an FA chain to produce palmitate, the primary saturated fatty acid precursor for more complex lipids [97]. Studies have shown that the overexpression of these enzymes is a common feature found in numerous cancer types; particularly, FASN and ACLY overexpression was reported in breast, prostate, colorectal, and renal carcinoma [98,99,100,101]. Besides increased FA synthesis and uptake, in cancer cells, fatty acid oxidation (FAO) enzymes are overexpressed and impact the energy production as well as the lipid metabolism [102].

When de novo lipogenesis is increased, the cancer cell membrane exhibits an enhanced short-chain FA (SC-FA) content which makes the membrane more densely packed and therefore less permeable to anticancer agents [103]. Thus, targeting the de novo lipogenesis in cancer cells is currently recognized as a successful therapeutic strategy [104,105]. Indeed, in SW620 colon cancer cells, treatment with DHA reduced the de novo synthesis of cholesterol [106]. In parallel, the increase in FA cellular uptake may also be implicated in the proliferation of cancer cells. In this regard, a recent study reports that DHA treatment can also inhibit cholesterol transport from the cellular membranes to the endoplasmic reticulum (ER) in CaCo2 colon cancer cells via an unknown mechanism [107]. A more complex mechanism for DHA anticancer effect was described after in vitro and in vivo experiments in androgen-responsive LNCaP, castration-resistant C4–2 and 22Rv1 prostate cancer cell lines, PCa castration-resistant organoids, and prostate cancer xenografts [108]. The data revealed that DHA has the ability to accumulate into lipid droplets as triacylglycerol and cholesterol esters, thus increasing the unsaturation of the phospholipid acyl chain and altering the phospholipid ratio, a known trigger of ER stress [108]. Moreover, DHA decreased SREBP transcriptional programming, which, in turn, led to a decreased expression of FASN and de novo lipogenesis [108].

**Figure 7:**
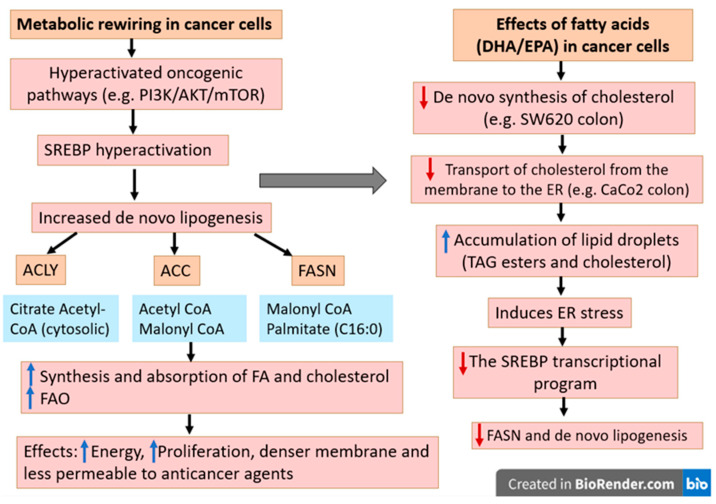
Modulation of lipid metabolism in cancer: therapeutic targets of n-3 fatty acids. ACC: Acetyl-CoA carboxylase; ACYL: acyl group; AKT: Protein kinase B; CaCo2: Caco-2 human colorectal adenocarcinoma cell line; CoA: Coenzyme A; DHA: Docosahexaenoic acid; EPA: Eicosapentaenoic acid; ER: Endoplasmic reticulum; FAO: Fatty acid oxidation; FASN: Fatty acid synthase; mTOR: Mechanistic Target of Rapamycin; PI3K: Phosphoinositide 3-kinase; SREBP: Sterol regulatory element-binding protein; SW620: Human colorectal adenocarcinoma cell line; TAGs: Triacylglycerols (Created in BioRender. Bătrîna, O. (2026) https://BioRender.com/eju32a6).

Taken together, all these studies suggest that the use of FA conjugated with specific pharmacological inhibitors or the use of certain FAs, such as DHA or EPA, that have the ability to target enzymes involved in the lipid metabolism of cancer cells are worthy anticancer therapeutic strategies that may be studied in depth for their ability to inhibit tumor progression (Figure 7).

#### 5.2.2. Membrane Remodeling and Lipid Raft Disruption

**Figure 8:**
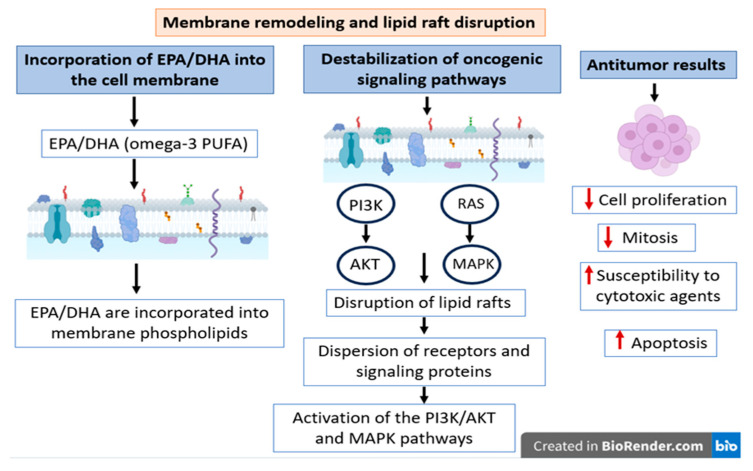
Membrane lipid remodeling and antitumor effects. AKT: Protein kinase B; DHA: Docosahexaenoic acid; EPA: Eicosapentaenoic acid; MAPK: Mitogen-Activated Protein Kinase; PI3K: Phosphoinositide 3-kinase; PUFAs: Polyunsaturated Fatty Acids; RAS: Rat Sarcoma virus protein (Created in BioRender. Bătrîna, O. (2026) https://BioRender.com/a5nhejq).

The use of FA conjugates is a promising strategy to modulate the lipid metabolism in cancer cells. Particularly, FA conjugates containing PUFAs or specific MUFAs, are known to interact with cell membranes, subsequently inducing structural remodeling and disrupting lipid rafts [109,110] (Figure 8).

Lipid rafts are dynamic cell membrane cholesterol- and sphingolipid-rich microdomains that play crucial roles in organizing signal transduction pathways, cell adhesion, and membrane trafficking. These rafts often serve as platforms for various oncogenic signaling molecules, such as those involved in the PI3K/AKT and RAS pathways, which are vital for cancer cell survival and proliferation [111]. Due to their high degree of unsaturation and structural flexibility, EPA and DHA, two omega-3 PUFAs, can be incorporated in cell membranes; in turn, due to the presence of cis-double bonds in their structure, this process increases the overall membrane fluidity and disrupts the tight packing of lipids that characterize lipid rafts, leading to their dissolution [112]. As a consequence, the clustering and activity of raft-associated receptors and downstream pro-survival signaling pathways, including PI3K/Akt and MAPK pathways, are destabilized [112]. Changes in membrane PUFA content influence receptor clustering, signal transduction and also the balance of eicosanoid synthesis by reducing AA-derived prostaglandins and thromboxanes in favor of EPA/DHA-derived mediators [113,114]. In vitro studies revealed that EPA and DHA can decrease cell viability and induce apoptosis in MDA-MB-231 breast cancer cells [115]. This effect was attributed to EPA’s and DHA’s ability to influence lipid raft composition by decreasing their content in sphingomyelin, cholesterol, and diacylglycerol while increasing the ceramide levels; additionally, they also caused a marked decrease in the EGFR levels and an increased phosphorylation of both EGFR and p38 MAPK [115]. As revealed by the group of Grimm et al., DHA-induced membrane cholesterol shifts from raft to non-raft domains in SH-SY5Y cells [116]. Other recent in vitro and ex vivo studies suggest that DHA and EPA have the ability to displace proteins in or out of the lipid rafts. In MDA-MB-231 breast cancer cells, both DHA and EPA exhibited a preventative effect against metastasis by disrupting the lipid raft domains and partially displacing CXCR4, a chemokine receptor involved in metastasis; this effect was not reported when stearic acid was tested [117]. In rat neural stem cells, LC-PUFAs DHA, but not AA, directly disrupted membrane microdomain compositions and profoundly affected the GFR and connexin 43 localization in lipid rafts [118]. In contrast, Shaikh et al. demonstrated that DHA and EPA do not directly modify lipid rafts but rather incorporate into non-rafts and modified the lateral organization and conformation of membrane proteins [119]. Specifically, DHA and EPA selectively altered major histocompatibility complex (MHC) class I lateral organization, not by inducing changes to its conformation but by increasing total surface levels relative to bovine serum albumin [119]. An altered membrane composition can translate into reduced cell proliferation and enhanced susceptibility to cytotoxic attacks. In vitro studies have reported decreased mitosis and tumor growth with increased LC-PUFA content, while in vivo clinical correlative studies report that higher marine-derived FA food intake or omega-3 PUFA increased cancer survival rates and decreased the risk of additional cancer events and all-cause mortality [120,121,122].

#### 5.2.3. Oxidative Stress

**Figure 9:**
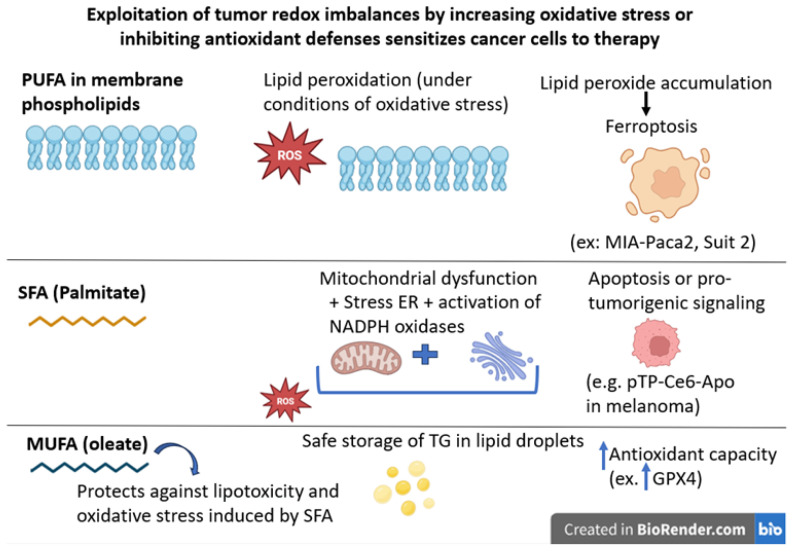
Induction of oxidative stress by fatty acids and effects on cancer cell survival. ER: Endoplasmic reticulum; GPX4: Glutathione peroxidase 4; MIA PaCa-2: Human pancreatic carcinoma cell line; MUFAs: Monounsaturated fatty acids; NADPH: Nicotinamide adenine dinucleotide phosphate; PTP–Ce6–Apo: Protein tyrosine phosphatase–chlorin e6–apolipoprotein conjugate; PUFAs: Polyunsaturated fatty acids; SFAs: Saturated fatty acids; SUIT-2: Human pancreatic adenocarcinoma cell line; TG: Triacylglycerols (Created in BioRender. Bătrîna, O. (2026) https://BioRender.com/xnguaek).

Exploiting tumor redox imbalances, either by triggering intracellular oxidative stress or by disabling antioxidant defenses, has become an important strategy to sensitize cancer cells to chemotherapy. This approach favors FA-based formulations due to their ability to be engineered for redox-sensitive release and to permit delivery of redox modulators or metabolic inhibitors that disturb the tumor protective pathways [123] (Figure 9).

Among FAs, PUFAs are very susceptible to oxidative damage due to the presence of multiple double bonds. In oxidative stress conditions, when incorporated into membrane phospholipids, PUFAs can undergo lipid peroxidation, and the accumulation of such lipid peroxides may trigger ferroptosis, a regulated type of cell death induced by iron-dependent lipid peroxidation [124]. This mechanism was demonstrated in MIA-Paca2 and Suit 2 pancreatic cancer cells, where linoleic acid (LA) and α-linolenic acid (ALA) increased lipid peroxidation, induced ferroptosis and suppressed tumor growth [10]. In contrast, SFA palmitate often promotes oxidative stress through mechanisms involving mitochondrial dysfunction, endoplasmic reticulum (ER) stress, and activation of NADPH oxidases, which can either trigger apoptosis or, at sublethal levels, support pro-tumorigenic signaling pathways [125]. In another study that used LC-SFAs, adipocytes loaded with a glutathione (GSH)-responsive palmitic acid–conjugated triptolide derivative (pTP) and Ce6 photosensitizer (pTP-Ce6-Apo) significantly inhibited melanoma growth and metastasis [90]. The release of pTP and Ce6 was triggered by intracellular GSH-induced lipolysis combined with laser irradiation and lead to increased ROS production and endoplasmic reticulum (ER) stress [90]. On the other hand, oleate, an MUFA, acts as a protective agent that buffers against SFA-induced lipotoxicity and oxidative stress by promoting safe triglyceride storage in lipid droplets and enhancing the cellular antioxidant capacity, including the upregulation of glutathione peroxidase 4 (GPX4) [125].

The development of FA conjugates with redox-responsive linkers can increase tumor vulnerability against conventional anticancer agents. In this regard, conjugated FAs, such (10*E*,12*Z*)-octadecadienoic acid and α-eleostearic acid (ESA), induced the chaperone-mediated autophagic (CMA) degradation of the key ferroptosis inhibitor glutathione peroxidase 4 (GPX4), the generation of mitochondrial ROS and lipid peroxides, and ultimately ferroptosis in HT1080 fibrosarcoma and A549 lung cancer cell lines; mitochondrial ROS proved to be sufficient and necessary for CMA-dependent degradation of GPX4 [126].

#### 5.2.4. Ferroptosis Induction

**Figure 10:**
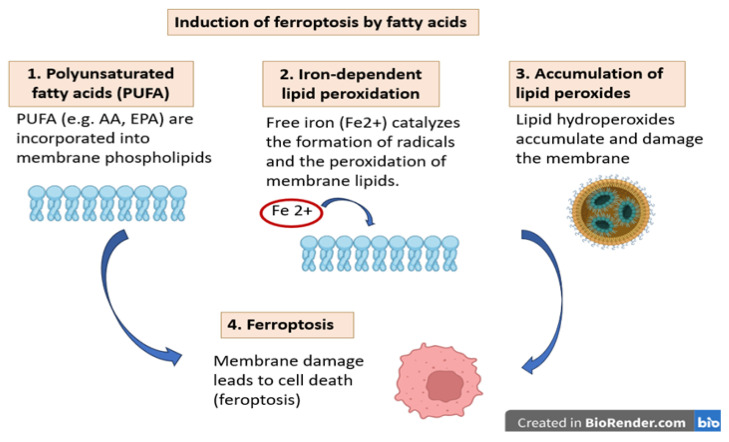
Induction of ferroptosis by lipid peroxide accumulation and cellular redox imbalance. AA: Arachidonic acid; EPA: Eicosapentaenoic acid; Fe^2+^: Ferrous iron; PUFAs: Polyunsaturated fatty acids (Created in BioRender. Bătrîna, O. (2026) https://BioRender.com/zwcgg4l).

Ferroptosis is a distinct, regulated form of cell death characterized by the iron-dependent accumulation of lipid hydroperoxides which compromise membrane integrity. This is a separate process that differs from apoptosis, necroptosis, and other canonical cell-death programs in both mechanistic and morphological ways [127,128]. Cell sensitivity to ferroptosis is influenced by the levels of peroxidizable PUFAs within its membranes and associated lipid metabolic enzymes [129]. The susceptibility of cells to ferroptosis is hence governed by a delicate balance among PUFA availability, the iron-catalyzed radical chemistry, and the cellular antioxidant defense mechanisms, primarily the glutathione-GPX4 axis and the FSP1-ubiquinol system [128,130,131] (Figure 10).

Given that PUFAs are preferred substrates for lipid peroxidation (due to the weak double bonds) and are modifiable components of the cellular lipidome, therapeutic strategies that target the PUFA supply, enzymatic processing (desaturation/elongation), incorporation into phospholipids, or their intracellular trafficking can be viewed as promising strategies to modulate ferroptosis in cancer treatment [132,133]. Mechanistically, PUFA-driven ferroptosis requires the generation of PUFA-containing phospholipids, a process dependent on fatty-acyl–CoA ligases, members of the ACSL family, and acyltransferases like LPCATs [131]. Also essential to this process is the existence of an oxidative environment that drives hydrogen abstraction and peroxyl radical chain reactions, mediated by lipoxygenases and non-enzymatic Fenton chemical reactions in the presence of labile iron [128,131]. The capacity of PUFAs to induce ferroptosis is not uniform, as both their chain length and the number and configuration of double bonds significantly impact peroxidation kinetics and biological outcomes. Studies have shown that AA and EPA can be particularly effective at sensitizing certain cancer cells to ferroptosis [18,134]. However, DHA has shown variable and sometimes weaker effects, depending on cell context and experimental conditions [18,134,135]. Conjugated PUFAs, such as ESA, are potent ferroptosis inducers; ESA activity is linked to its distinct incorporation into cellular lipids and subsequent peroxidative reactivity, with ACSL1-dependent esterification identified as a key step in promoting lipid peroxidation [135]. Furthermore, the omega-6 PUFA metabolites have also been shown to drive ferroptosis in non-neoplastic contexts, emphasizing that dietary enrichment with omega-6 PUFA may increase the pro-ferroptotic signaling in some settings [133,136].

The subcellular trafficking of PUFAs, their sequestration into neutral lipid droplets, and the regulated mobilization of those deposits are critical determinants of ferroptosis. While the initial stress responses may lead to the accumulation of lipid droplets, the lipolytic or lipophagic breakdown of these droplets releases PUFA-containing acyl chains; such chains can be re-esterified into membrane phospholipids, thereby increasing the ferroptotic vulnerability [132]. Conversely, mechanisms that sequester PUFAs away from peroxidation-prone membranes, either by storage in triacylglycerols or by limiting their acylation into phospholipids, can protect cells against ferroptosis. This adaptive principle has been reported in cells with altered oxidative phosphorylation or in tumor environments where PUFA trafficking is regulated to reduce membrane unsaturation [137,138]. Consequently, therapeutic approaches that aim to promote the release of PUFAs from storage into the phospholipid pools or to prevent their sequestration are mechanistically attractive to amplify ferroptotic responses.

While PUFAs are central to ferroptosis, there are several other classes of FAs that, when tested alone or conjugated with drugs, exert significant influence on ferroptosis. The impact of these FA often stems from their ability to modulate the lipid metabolism, interact with key ferroptosis regulatory pathways, or alter the cellular redox state [127,128,139]. Contrary to PUFA’s effect on ferroptosis, exogenous OA (C18:1) MUFA induced a ferroptosis-resistant cell state by suppressing the accumulation of lipid peroxides and decreasing the levels of oxidizable PUFAs [139]. This protective effect was attributed to MUFA incorporation into phospholipids via ACSL3, which displaced PUFAs from membrane phospholipids, consequently reducing the substrate’s availability for lipid peroxidation [140]. This finding reveals that the fluctuations in the extracellular lipid availability and the competitive incorporation of PUFAs and MUFAs into the cellular membrane play an important role in determining the cellular state and its susceptibility to ferroptosis.

#### 5.2.5. Anti-Inflammatory and Immune-Modulatory Effects

**Figure 11:**
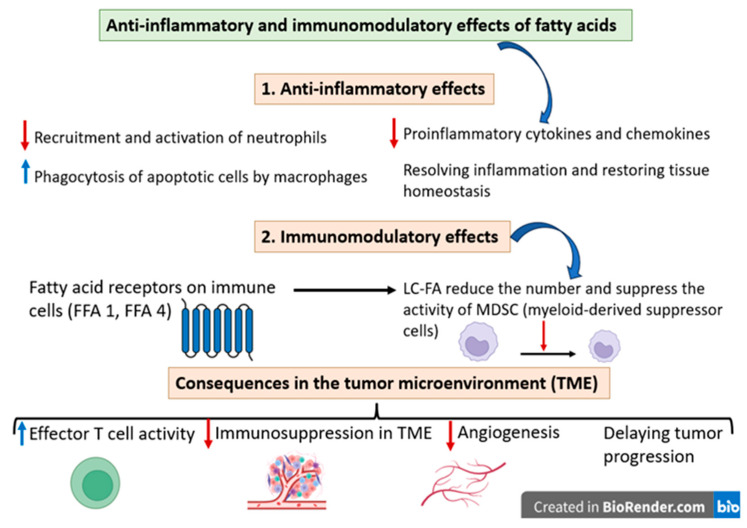
Anti-inflammatory and immunomodulatory effects of fatty acids in regulating the tumor microenvironment. FFAs: Free Fatty Acids; LC-FAs: Long-chain fatty acids; MDSCs: Myeloid-derived suppressor cells; TME: Tumor Microenvironment (Created in BioRender. Bătrîna, O. (2026) https://BioRender.com/z8wxhv1).

Studies have shown that inflammation plays a crucial role in tumor progression, angiogenesis, and immune suppression within the tumor microenvironment; hence, modulation of the inflammatory signaling by FA and FA conjugates may represent another anticancer therapeutic strategy (Figure 11).

PUFAs, such as EPA and DHA, are recognized for their anti-inflammatory properties [141,142]. More specifically, EPA and DHA are enzymatic precursors of specialized pro-resolving mediators (SPMs), including resolvins, protectins, and maresins, that actively terminate inflammation and promote the restoration of tissue homeostasis [143,144]. SPMs derived from EPA and DHA act as agonists for specific G-protein-coupled receptors, such as CMKLR1, BLT1, ALX/FPR2, GPR32, and limit neutrophil recruitment and activation, reduce pro-inflammatory cytokine and chemokine production, and accelerate macrophage-mediated clearance of debris and apoptotic cells [143,144]. Resolvins, derived from omega-3 FAs, can suppress several cancer-related molecular pathways, including the activation of transcription factors in tumor cells and the TME, inflammatory cell infiltration, and the production of cytokines and chemokines [145]. Studies focusing on inflammation-driven malignancies, particularly gastrointestinal and breast cancers, emphasize that these SPMs can mitigate the chronic, unresolved inflammation that contributes to tumor initiation and progression [122,146,147]. Systematic appraisal of randomized trials also supports the hypothesis that EPA/DHA supplementation can decrease the inflammatory biomarkers in certain patient groups; however, this effect varies according to the population and the baseline inflammatory status [148,149]. Supplementation with omega-3 PUFA has been shown to reduce inflammatory factors like interleukin-6 (IL-6) and tumor necrosis factor-alpha (TNF-α) in cancer patients [150]. In contrast, omega-6 PUFAs play a dual role, exhibiting both pro-inflammatory and anti-inflammatory effects depending on the specific FA and cancer context [151]. In a murine model of pulmonary squamous cell carcinoma, mice that were fed a diet rich in omega-6 PUFA showed an increase in pro-inflammatory markers, tumor proliferation, angiogenesis and a decreased expression of pro-apoptotic proteins [152]. By contrast, a meta-analysis involving 1252 patients, healthy or carrying inflammatory diseases, concluded that dietary omega-6 PUFAs do not increase the risk of inflammation or chronic inflammatory conditions [153]. Collectively, the ability of EPA/DHA to shift the eicosanoid biology away from AA-derived pro-inflammatory mediators toward SPMs provides a biologically plausible route by which these FA could reduce tumor-promoting inflammation and modulate the tumor microenvironment in ways favorable to therapy [113,143,144]. According to two separate studies, punicic acid, a potent omega-5 PUFA, is able to reduce the expression of pro-inflammatory cytokines/chemokines such as IL-2, IL-6, IL-12, IL-17, IP-10, MIP-1α, MIP-1β, MCP-1 and TNF-α in breast, colon, liver and prostate cancer cell lines [154,155].

The presence of FA receptors in immune cells, such as FFA1 and FFA4, contributes to understanding their role as anti-inflammatory or pro-inflammatory molecules and their respective intracellular mechanisms [156]. FAs can directly modulate the function of immune cells; LC-FAs have been shown to reduce the number and suppress the activity of myeloid-derived suppressor cells (MDSCs) both in vitro and in vivo [157]. MDSCs are key components of the immunosuppressive TME and play a major role in tumor immune evasion [158]. A study showed that by inhibiting MDSCs, LC-FAs (oleic acid:palmitic acid:stearic acid = 2:2:1) can delay tumor progression in allograft models of RM-1 prostate and LLC lung carcinoma cancer cells [157].

In summary, FAs and their conjugates modulate both the inflammatory processes and the immune responses within the tumor microenvironment through diverse mechanisms, including the generation of SPMs, direct effects on the immune cell function (like MDSCs and T-cells), and the reprogramming of cancer cell metabolism. These multifaceted roles highlight their potential as tools to boost cancer immunotherapy efficacy and as therapeutic targets in oncology.

#### 5.2.6. Regulation of Cell Cycle, Proliferation and Apoptosis

FAs can directly influence tumor cell proliferation by modulating the key regulators of the cell cycle and apoptosis pathways [159]. Different classes of FAs, and in particular, LC-PUFAs, can inhibit cancer cell growth by inducing cell cycle arrest, suppressing proliferation signaling pathways, and activating programmed cell death mechanisms [160,161,162]. These effects are mediated through the modulation of cyclins, cyclin-dependent kinases (CDKs), tumor suppressor proteins such as p53, and apoptotic regulators, including caspases and Bcl-2 family proteins; these representative examples of FA-mediated mechanisms are summarized in Table 8.

As illustrated in Table 5, FAs can interfere with multiple signaling pathways involved in cancer cell survival, tumor growth, progression and metastasis. Depending on their chain length, degree of unsaturation, and molecular structure, FAs are able to exert diverse biological effects in tumor cells. Polyunsaturated FAs (PUFAs), particularly DHA and EPA, are the most frequently studied and consistently demonstrate antiproliferative effects through mechanisms such as suppression of the AKT pathway, mitochondrial dysfunction, oxidative stress induction, lipid peroxidation that seems to lead to ferroptosis, apoptosis and even pyroptosis. Several studies also report on PUFA’s ability to modulate cyclins and cyclin-dependent kinases, thus leading to cell cycle arrest, most commonly at the G0/G1 or G2/M checkpoints. Furthermore, SFAs have also shown cytotoxic activity in cancer cell lines through mitochondrial dysfunction and caspase activation, modulation of ROS production and increased phosphorylation of protein pathways that lead to apoptosis. By contrast, through various mechanisms, the majority of MUFAs increase cell proliferation and migration in cancer cells. Overall, the studies presented in Table 5 indicate that FA per se can influence numerous hallmarks of cancer, including proliferation, survival, and programmed cell death—data that support their potential role as complementary therapeutic agents.

#### 5.2.7. Sensitization to Anticancer Therapies

Unfortunately, a major limitation of the chemotherapy efficacy is the occurrence of cancer cell resistance to drugs. In this context, FAs, and notably LC-PUFAs, are viewed as chemosensitizing agents capable of reversing multidrug resistance (MDR) by partially elucidated pleiotropic mechanisms [207]. Specifically, Cha et al. revealed that omega-3 LC-PUFA DHA in combination with arabinosylcytosine can prolong the life expectancy of mice bearing L1210 leukemia [208]. In C6 and U87-MG glioblastoma cells, the conjugation with LA, OA and palmitic acid increased the cytotoxic effect of temozolomide; these results were supported by further in vivo experiments that revealed a 1.6-fold improvement in the survival rate of rat glioblastoma models [209]. Similarly, DOX associated with non-toxic doses of DHA was more cytotoxic compared with DOX alone in A-172 glioblastoma cells [210]. The same DOX co-treatment with DHA was able to inhibit the growth and invasion of MCF-7 breast cancer cells that were previously resistant to DOX alone [211]. In another breast cancer cell line, MDA-MB-231, DHA was able to increase the cytotoxic effect of taxanes, PTX and docetaxel, thus suggesting the presence of a strong synergistic cytotoxic effect by regulating the involved oncoprotein expression [212]. In SW480 colon and PC3 prostate cancer cells, the conjugation of ciprofloxacin with various types of SFAs, MUFAs and PUFAs increased the resulting anticancer activity; the most promising inhibitory effects in SW480 cells were exerted by derivatives conjugated with short (sorbic acid, IC_50_ = 20.1 ± 2.1 μM), middle (geranic acid, IC_50_ = 32.8 ± 2.4 μM) and long (DHA, IC_50_ = 26.2 ± 24.3 μM) PUFA hydrocarbon chains, while in PC3 cells the highest potency was recorded for MUFA OA (IC_50_ = 7.7 ± 2.1 μM) and PUFA sorbic acid (IC_50_ = 11.7 ± 1.8 μM) [213]. EPA, after its successful incorporation into the cell membrane, increased the sensitivity of COLO 320 DM colon cancer cells to 5-Fluorouracil and oxaliplatin [214]. Similarly, the inhibition of Akt by EPA restored the response to tamoxifen in MCF-7 breast cancer cells [181].

Another study reported the use of FAs to extend the therapeutic spectrum and to overcome the de novo acquired resistance to anticancer drugs. In As_2_O_3_-resistant solid tumor cells, the combined treatment with As_2_O_3_ and DHA significantly reduced the viability of 7 of the 10 solid tumors tested, with no cytotoxic effect against normal skin fibroblasts [215]. There are several other studies that report on LC-PUFA’s ability to overcome MDR, an effect that occurs most probably due to their ability to alter membrane fluidity, displace cholesterol from lipid rafts, and by inhibiting the endogenous cholesterol synthesis [207,216], as previously described. As described by Das et al., the LC-PUFA incorporation is correlated with an increase in the drug uptake/efflux ratio [217]. The changes induced by LC-PUFAs were reported to be more noticeable in chemoresistant cells compared to chemosensitive cells [218]. P-glycoprotein (Pgp) overexpression is a prevalent event in cells with MDR; in HT9/MDR colon cancer cells, DHA and EPA, after their incorporation in the lipid rafts, were able to reduce the amount of membrane/lipid raft-Pgp and to restore cell sensitivity to DOX and irinotecan [216].

### 5.3. FAs as Adjuvants in Cancer Therapy

Among the numerous PUFAs, EPA and DHA have been extensively investigated as adjuvants in cancer therapy due to their potential to modulate inflammation, oxidative stress, and tumor cell metabolism [219,220], as previously discussed. Beyond their direct effect in cancer cells, there is also clinical and epidemiologic evidence for their use; however, the available data are heterogeneous and tumor- and context-dependent [122,147]. The nutritional supplementation with EPA-rich formulations has been reported to stabilize body weight, reduce inflammation-related markers, and improve nutritional status in certain patient population [221]. Translational clinical evaluation, including pilot and phase II studies using fish-oil formulations (including Omegaven^®^ and other EPA/DHA preparations) combined with conventional chemotherapy, have reported improved tolerability and nutritional endpoints, and, in some cases, improvements in clinical outcomes that warrant further randomized testing [122,222,223]. These data support the hypothesis that EPA/DHA may act as true adjuvants by both inducing the biologic sensitization of tumor cells and ameliorating the treatment-related systemic effects that limit dose intensity. Cancer cachexia is a frequent and important syndrome in terms of prognosis. Trials and research studies have examined whether omega-3 LC-PUFAs can prevent or attenuate cancer-related weight loss and inflammation. Systematic studies in gastrointestinal and lung cancer cohorts indicate that omega-3 PUFAs supplementation combined with anticancer therapies may reduce anorexia/cachexia, as well as increase patient weight and lean mass, while reducing inflammatory markers [122,146]. Moreover, the evidence suggest that benefits are more pronounced in older patients (≥67 years) and in those with a baseline body weight ≤ 60 kg, thus highlighting the importance of patient age and nutritional status when omega-3s are used as adjuvants [224]. Other studies of breast and other cancer cohorts suggest that omega-3 PUFAs administered in combination with chemotherapy and radiotherapy can reduce pain and improve tolerability; however, these effects are heterogeneous and require standardization [121,122,223].

It is known that both the dose and the specific formulation are able to influence bioavailability, RBC incorporation, as well as other biological effects. In the case of omega-3 LC-PUFAs, randomized dose-response studies show that the erythrocyte omega-3 content increases with intake but that the magnitude of change varies according to the baseline status and dose [225]. The type of the formulation also influences the omega-3 performance; ethyl-ester preparations, triglyceride (TG) or re-esterified TG formulations are available and used in clinical studies. Their pharmacokinetics and RBC incorporation kinetics differ and may be influenced by co-ingested fat and digestive status [223,225,226]. Trial protocols should record formulation, dose and adherence, and also consider using the RBC omega-3 index as a pharmacodynamic measure to confirm target membrane incorporation [223,225,226]. Taken together, further well-controlled clinical studies are required to clarify the therapeutic role of omega-3 in oncology.

Beyond the dietary omega-3 supplementation, there are other FA–drug conjugates that have also demonstrated adjuvant anticancer potential. A notable example is DHA–paclitaxel (Taxoprexin), presented in Table 7. Even though early clinical phase I and II studies demonstrated the anticancer activity of this conjugate in several solid tumors, illustrating the potential of FA conjugation to improve the pharmacokinetics and therapeutic response compared with the parent drug, phase III studies failed to show enhanced effects over pure dacarbazine [227,228].

Overall, FAs can act as multifunctional adjuvants in cancer therapy through their pleiotropic effects but also by improving drug delivery. Although promising, the clinical efficacy of FA-based adjuvant strategies remains dependent on numerous parameters such as FA type, dosage, formulation, tumor context, patient age and nutritional status, thus highlighting the need for further controlled clinical studies.

## 6. Future Directions

FA-based strategies are flexible tools in oncology that allow the improvement of drug delivery, pharmacokinetics and therapeutic efficiency and selectivity through FA–drug conjugation and FA-containing lipid nanocarriers. The evidence presented in this review highlights the ability of FA moieties to improve the anticancer activity of the resulting compound by modifying its membrane permeability, its binding to serum albumin, as well as its lymphatic transport and intracellular drug activation. However, despite numerous advances in the field of FA use in oncology, there are several research directions that remain critical to be further approached and understood in order to be able to convert FA-based technologies into clinically effective therapeutic strategies.

One important future path involves the rational molecular design of FA-drug conjugates. Although many current studies state that parameters such as FA chain length, degree of unsaturation, and linker chemistry can significantly influence their pharmacokinetic behavior, a thorough understanding of how these structural characteristics modify biodistribution, tumor uptake, and enzymatic activation is yet to be fully elucidated. In this regard, future studies should focus on identifying the exact structure–activity relationships that can produce FA moieties and linkers with predictable release kinetics and improved tumor selectivity.

Another promising research direction is represented by the development of FA-based delivery systems that are stimuli-responsive. TME has unique biochemical traits such as elevated ROS levels, altered pH and increased levels of glutathione. FA-modified prodrugs/FA nanocarriers stimuli-sensitive/responsive linkers may be able to allow a selective activation of drugs within the tumor sites while reducing the systemic exposure. These strategies could further increase the therapeutic index of those cytotoxic agents while also improving safety profiles.

More advanced FA-based nanoplatforms are also made possible by developments in the lipid nanotechnology. Hybrid systems that combine FA-modified lipids with polymers, peptides, or targeting ligands might facilitate an improved control over biodistribution, immune interactions, and cellular uptake mechanisms. Specifically, more accurate tumor targeting may be made possible by receptor-targeted nanocarriers that take advantage of tumor-associated transporters involved in the lipid metabolism, such as lipoprotein receptors or FA transport proteins.

Another growing area of interest is the biological activity of FAs themselves. As presented in this review, beyond their use as delivery systems, there are several FAs, mostly but not limited to PUFAs, that can alter tumor cell metabolism, oxidative stress pathways, and cell death mechanisms. The intrinsic anticancer characteristics of particular FAs may be combined with FA-driven delivery mechanisms in order to create dual-function platforms that function as both carriers and biological active agents.

Despite promising preclinical results, the clinical translation of FA-based systems is still limited, underscoring the need for additional pharmacokinetic, toxicological and manufacturing R&D. To guarantee regulatory approval and clinical applicability, practical issues like large-scale synthesis, formulation stability, and reproducibility must be addressed. To confirm the safety and therapeutic advantages of FA-derived prodrugs and lipid nanocarriers across various cancer types, more thorough in vivo research and clinical trials will be necessary.

Lastly, integrated multidisciplinary approaches that combine medicinal chemistry, nanotechnology, lipid biology, and systems pharmacology are beneficial for future advancements. Anticipating interactions with biological membranes, serum proteins, and metabolic pathways, developments in computational modeling and lipidomics will further aid in the logical design of FA-based therapeutic systems.

When combined, these FA-based approaches offer a promising and rapidly growing field for the development of anticancer drugs. Further studies of lipid-mediated biological mechanisms, nanocarrier engineering, and FA–drug conjugation chemistry will increase the therapeutic potential of FA and support the development of more potent and targeted cancer therapies.

## 7. Conclusions

FAs are versatile structures with intrinsic activity and numerous effects on cancer cells that can be used for the development of new anticancer strategies, acting as structural components in lipid-based delivery systems as well as chemical modifiers in prodrug design. Drug physicochemical properties, pharmacokinetics, and biodistribution can be rationally modulated due to their endogenous origin, biocompatibility, and structural diversity. FAs can improve drug systemic circulation time, facilitate its lymphatic transport, increase its membrane permeability, and encourage the accumulation of anticancer compounds in tumors through the covalent conjugation to therapeutic agents or incorporation into lipid nanocarriers. Studies revealed that FA–drug conjugation is a useful strategy to maximize the pharmacological efficacy of anticancer drugs. The controlled release of the parent drug is made possible by customizing the resulting lipophilicity, stability, and enzymatic activation profiles through the selection of suitable FA moieties and linker chemistry. Ester, amide, carbamate, carbonate, and thioester linkages provide different levels of stability and release kinetics, being flexible tools for the design of lipophilic prodrugs with predictable pharmacokinetic behavior. Simultaneously, FA-based lipid nanocarriers, such as liposomes, LNPs, self-assembling LAPs, and SNEDDS, have shown great potential to enhance drug delivery in oncology. Through processes like albumin binding, receptor-mediated uptake, and through the EPR effect, these systems can protect the therapeutic payloads from premature degradation, improve the solubilization of hydrophobic drugs, and increase tumor exposure to drugs. Among these platforms, liposomal and LNP systems offer flexible and clinically proven delivery frameworks, while self-assembling LAPs provide exceptionally high drug loading and controlled intracellular activation. In addition to their function as delivery components, some FAs, particularly PUFAs, have intrinsic biological activities that could affect cancer progression and its response to therapy. These activities include the regulation of cell death pathways like ferroptosis, apoptosis, oxidative stress, and lipid metabolism. This dual functionality of FAs increases their therapeutic relevance in oncology. Overall, the combination of FA chemistry, nanotechnology, and drug design represents a powerful platform to improve effectiveness and selectivity in cancer therapy. The role of FAs in the next-generation oncological therapies will be further strengthened by the ongoing research in lipid–tumor interactions, FA-based prodrug development and lipid nanocarrier engineering.

## Figures and Tables

**Table 1:** Synthetic methods for FA–drug conjugation: reaction conditions, mechanisms, advantages, and limitations.

Method	Conditions	Mechanistic Feature/Reactivity Driver	Advantage	Limitation	Reference
Acid chlorides/anhydrides	Strictly anhydrous conditions, usually in the presence of a base or acid scavenger	FA carboxyl groups are converted into highly activated acyl derivatives. The drug hydroxyl group attacks the electrophilic carbonyl carbon, forming a tetrahedral intermediate, followed by elimination of chloride or carboxylate as the leaving group. The strong electrophilicity of acyl chlorides enables rapid acyl transfer but increases the risk of side reactions.	High yield FA chain variability does not hinder acid chloride/anhydride formation → fine-tuning of conjugate physicochemical properties (lipophilicity and membrane affinity)	Stringent control of reaction parameters Acidic by-products may promote degradation or side reactions Limited selectivity in multifunctional drugs Possible loss of FA’s carboxylate group	[22]
The Steglich Esterification (Carbodiimide)	Mild, near-neutral, anhydrous conditions	Carbodiimide activation converts the FA carboxyl group into an O-acylisourea intermediate. DMAP can generate a more reactive acyl-pyridinium species, which is attacked by the drug hydroxyl group to form the ester. Reactivity depends on the accessibility of the FA carboxyl group and the nucleophilicity/steric environment of the drug hydroxyl group.	Tolerant functional group No strong acids/high temperatures are required → the reaction is highly compatible with molecules containing sensitive or multifunctional scaffolds	Urea by-products Selectivity may be impacted when the drug contains multiple nucleophilic sites	[23]
T3P-mediated coupling	Mild conditions, commonly with organic base in aprotic solvent	T3P activates the FA carboxyl group through mixed phosphonic anhydride intermediates. These intermediates increase carbonyl electrophilicity and promote nucleophilic attack by alcohols or amines, enabling ester or amide bond formation. Water-soluble phosphorus-containing by-products simplify purification.	Clean, predictable and controllable reactions Broad compatibility with diverse substrates Efficient for ester and amide formation Suitable for sensitive molecules due to mild activation.	High reagent cost Phosphorus-derived residues Reaction outcome depends on substrate solubility, steric accessibility, and nucleophile strength Long-chain or poorly soluble FA may decrease coupling efficiency	[24]
Lipase-catalyzed esterification	Non-aqueous or low-water organic medium; con-trolled water activity	The catalytic Ser-His-Asp triad forms an acyl-enzyme intermediate from the FA substrate. The alcohol-containing drug then attacks this intermediate, releasing the ester product. Substrate recognition is controlled by the enzyme active site, lid-domain dynamics, FA chain length, unsaturation, and diffusion of hydrophobic substrates.	High chemo-, regio-, and stereoselectivity; Avoids harsh conditions (strong acids, dehydrating agents) Minimal requirement for protective groups Suitable for sensitive or multi-functional drug molecules	Slow reaction rates High cost Complex downstream processing Loss/inhibition of activity	[25,26]

**Table 2:** Representative FA–drug linker strategies, target groups, advantages, and limitations.

Method/Linkage Type	Typical Reagents & Conditions	Target Group	Pros	Cons	Typical Release t^1/2^	Ref.
Amide coupling (EDCI/DMAP)	FA–COOH + EDCI + DMAP, anhydrous solvent, rt/overnight	Amine (e.g., aminoalcohols)	Robust coupling; good yields; high stability	Release usually slow (high stability)	Long	[57]
Amide via ester intermediate (methyl ester → aminolysis)	FA methyl ester + ethylenediamine, pyridine, reflux	Amine	Straightforward access to diamide/amine derivatives	Harsher conditions; not “prodrug-like” release	Long	[58]
Esterification on taxane C2′-OH (FA–taxane prodrug)	FA introduced at taxane C2′-OH (prodrug strategy)	Secondary OH (taxane)	Enables lipophilic FA–drug prodrug series	Hydrolysis rate depends on site/sterics	Medium (design-dependent)	[58]
ROS-labile linker in polymeric system (stimuli-responsive release)	Incorporation of ROS-sensitive linker in PLGA-based system	— (matrix-triggered)	Release can be biased to oxidative tumor microenvironment (TME)	Carrier complexity; release depends on ROS level	Trigger-dependent	[18]

**Table 3:** Physicochemical properties and biological performance of liposomal systems.

Liposomal Systems (DSPC/DOPE/Cholesterol ± FA)								
Size [nm]	PDI	Zeta Potential [mV]	Encapsulation Efficiency [%]	Drug Loading [% *w*/*w*]	Enzyme Triggered Release	Albumin Interaction	Biodistribution/Tumor Uptake	Ref.
≈40	N/A	“Negative”	>80	10	Not reported	Liposomes (PC:PS:OA) were 1.5–2 times more efficient for cellular uptake vs. albumin-FA complexes	High uptake in mammary tumor cells → leading to 50% higher DNA levels than controls	[70]
228.6 ± 2.6	0.107	≈−25 to −30	53.4% ± 1.1% − Ce6, 65.7% ± 2.1% − αPD-L1	5.3% − Ce6, 0.6% − αPD-L1	Photo-triggered ROS-responsive release	Not reported	Peritumoral injection + NIR (Photo-immunotherapy)	[71]
FA-EPA-Lip-QUE (pH 7.4): 106.4 ± 0.5 FA-EPA-Lip-QUE (pH 5.0): 128.1 ± 0.9 FA-EPA-Lip (blank): 96.0 ± 0.7	FA-EPA-Lip-QUE (pH 7.4): 0.25 ± 0.01 FA-EPA-Lip-QUE (pH 5.0): 0.32 ± 0.01 FA-EPA-Lip (blank): 0.18 ± 0.02	FA-EPA-Lip-QUE (pH 7.4): −30.6 ± 0.1 FA-EPA-Lip-QUE (pH 5.0): −24.3 ± 0.2 FA-EPA-Lip (blank): −27.4 ± 0.5	96.3 ± 0.1	~5.5	Not enzyme-responsive	Not reported	Enhanced cellular uptake in Folate Receptor (FR)-positive cells (HeLa and HepG2) via receptor-mediated endocytosis	[72]
DHA-PC:CHOL:TOCO: ~80 DHA-PC:TOCO: ~80 DHA-PC:DMPG:CHOL:TOCO: ~95 DHA-PC:DOTAP:CHOL:TOCO: ~90	DHA-PC:CHOL:TOCO: ~0.23 DHA-PC:TOCO: ~0.21 DHA-PC:DMPG:CHOL:TOCO: ~0.25 DHA-PC:DOTAP:CHOL:TOCO: ~0.24	DHA-PC:CHOL:TOCO): ~−22 lipid anionic DMPG: ~−40 lipid cationic DOTAP: ~+40	Not reported	20	Release triggered by external stimuli: heat or ultrasound	Not explicitly measured; pre-mixing strategy was used to prevent interference from biological components in vivo	Significantly enhanced tumor accumulation of DOX via pre-mixing	[73]
135.2 ± 3.4	0.182 ± 0.02	−24.8 ± 1.2	84.6 ± 2.1	5.4 ± 0.6	pH-dependent and diffusion-controlled release	High stability in serum (BSA/FBS) formulation maintained particle size and prevented drug leakage + minimal disruptive interaction with albumin	Significantly higher tumor accumulation and prolonged circulation time vs. free PTX Major accumulation in the tumor, followed by the liver and spleen	[74]
154 ± 4	0.19 ± 0.03	−41 ± 2	>90	22	Passive diffusion	Stabilized by PEGylation (DSPE-PEG2000) → minimized protein corona formation and prevention of aggregation → colloidal stability in protein-rich environments	High HCT116 and LS174T cellular internalization + cytoplasmic delivery of 5-FU, DHA, and EPA into cells Bypassed P-gp efflux → enhancing intracellular drug accumulation	[75]
163.3 ± 4.5	0.25 ± 0.01	+45.8 ± 1.2	96.6 ± 0.5	~3.5	N/A (passive diffusion and membrane fusion)	Stabilized by PEGylation (DSPE-PEG2000) → minimized protein corona formation + high encapsulation efficiency (>95%) → targeted intracellular delivery	Effective intracellular delivery to tumor cells Local acetate metabolization Minimal systemic exposure due to liposomal encapsulation and i.p. administration	[76]

**Table 4:** Physicochemical properties and biological performance of lipid nanoparticles.

LNPs								
Size [nm]	PDI	Zeta Potential [mV]	Encapsulation Efficiency [%]	Drug Loading [% *w*/*w*]	Enzyme Triggered Release	Albumin Interaction	Biodistribution/Tumor Uptake	Ref.
26.4 ± 1.2	0.17 ± 0.05	−14.8 ± 1.1	~100	25 ± 3	Rapid drug release → metabolization of FA core after receptor-mediated endocytosis FA also protected the payload from lysosomal degradation	Long blood circulation (2–4 days) and avoidance of non-specific protein adsorption or immune clearance.	Highly selective tumor accumulation via LDL receptor-mediated uptake	[77]
~100	0.12 to 0.15	−2 to +2	>90	~2	pH-responsive release; endosomal escape facilitated by ionizable lipids	Minimal/Controlled interaction due to PEGylation and spleen-selective engineering	Massive splenic accumulation Bypassed the liver; specifically targeting of T cells	[78]

**Table 5:** Physicochemical properties and biological performance of self-nanoemulsifying drug delivery systems.

SNEDDS								
Size [nm]	PDI	Zeta Potential [mV]	Encapsulation Efficiency [%]	Drug Loading [% *w*/*w*]	Enzyme Triggered Release	Albumin Interaction	Biodistribution/Tumor Uptake	Ref.
121.50 ± 0.17	0.140 ± 0.05	−12.3	~100	0.375 ± 0.12	Not reported	Not reported	Enhanced cytotoxicity and drug accumulation in MDA-MB-231 breast cancer cells	[79]
MCT-SNEDDS: 68.4 LCT-SNEDDS: 85.2	MCT-SNEDDS: 0.165 LCT-SNEDDS: 0.218	MCT-SNEDDS: +22.4 LCT-SNEDDS: +18.6	~100	1.2	N/A (Passive release/Solubilization)	Not explicitly studied; steric stabilization via nonionic surfactants (Tween 40) prevents protein interference, while cationic charge is optimized for mucoadhesion rather than plasma protein binding	Enhanced oral bioavailability via lymphatic transport; Superior mucoadhesion and intestinal absorption → significantly increased PTX plasma levels vs. pure drug (validated in rats)	[81]

**Table 6:** Physicochemical properties and biological performance of FA-modified lipid-based nanocarrier systems.

Self-Assembling LAPs								
Size [nm]	PDI	Zeta Potential [mV]	Encapsulation Efficiency [%]	Drug Loading [% *w*/*w*]	Enzyme Triggered Release	Albumin Interaction	Biodistribution/Tumor Uptake	Ref.
~100 to ~130	<0.15	−22.3 ± 1.5	99.5% ± 0.3	~42.5	Intracellular release via cathepsins B and L (lysosomal enzymes)	High affinity for serum albumin (BSA and HSA), forming a stable complex that acts as an albumin-based carrier, protecting SqGem from premature hydrolysis	10-fold increase in tumor drug concentration via long blood circulation (t^1/2^ 6.5 h) and the EPR effect	[83]
LLN (C12): 68.2 ± 1.8 nm. LPN (C16): 77.4 ± 2.1 nm. LSN (C18): 102.0 ± 4.6 nm.	LLN (C12): 0.14 ± 0.05 LPN (C16): 0.11 ± 0.02 LSN (C18): 0.18 ± 0.04	LLN (C12): −32.3 ± 2.4 LPN (C16): −36.2 ± 3.1 LSN (C18): −45.6 ± 4.2	~100	~82 to 87	Plasma enzyme-mediated hydrolysis of the amide bond (C12 > C16 > C18)	Formation of a “protein corona” with human serum albumin (HSA), leading to a slight increase in particle size. This binding protects the conjugate and enhances metabolic stability in plasma	Enhanced accumulation via the EPR effect; LFNs showed prolonged circulation and high concentration in the liver, spleen, and tumor. The system achieved deep penetration and strong inhibitory effects in 3D tumor spheroids (MCTS)	[84]
BFA C16: 124.9 BFA C18: 112.5 BFA C20: 119.8	BAC16: 0.17 BAC18: 0.13 BAC20: 0.15	−30	~100	>50	Release is mainly triggered by Redox/GSH and ROS (H2O2), which cleave the disulfide bonds	Not the main focus; the system uses DSPE-mPEG2k coating to prevent non-specific protein binding and compares its performance against the albumin-based benchmark Abraxane	The nano-assemblies showed prolonged blood circulation and high tumor-selective accumulation via the EPR effect, with tumor uptake increasing alongside the lipid chain length in the order BAC20 > BAC18 > BAC16	[85]

**Table 7:** FA–drug/FA-assisted anticancer constructs.

Construct	FA Moiety	Linker/Format	Model	Outcome (Pe Scurt)	Safety/PK Note	Ref.
DHA–PTX (Taxoprexin)	DHA	Ester at C2′ (taxane prodrug)	Mouse xenografts; clinical investigation mentioned	Antitumor activity in xenografts; lower systemic toxicity vs. parent taxoids; discussed as PTX conjugate in humans	FA–taxane prodrug series with DHA at C2′	[58]
PLGA nanoparticles (NPs) co-loading docetaxel + DHA (FA-assisted)	DHA	Co-encapsulation (NPs); release within 24 h described	HCT116 colon cancer cells	Encapsulation increased efficacy vs. free combo after 72 h; suggests protection of DHA and improved delivery	Carrier improved performance; empty NPs non-cytotoxic	[18]
Functional DHA/LA amide derivatives (anticancer FA-derivatives)	DHA, LA	Amides	MCF-7 breast cancer + normal fibroblasts	Cytotoxic on MCF-7; low toxicity in normal fibroblasts; apoptosis shown for top compounds	Compared conceptually with cisplatin selectivity/toxicity	[57]
Lipid metabolism targeting to sensitize chemotherapy (concept support for lipid/FA approaches)	—	Metabolism focus	Pancreatic cancer	Lipid metabolic reprogramming linked to progression/therapy response; targeting lipid metabolism can sensitize to chemotherapy	Supports rationale for lipid/FA-based distribution tuning	[57]

**Table 8:** Representative studies describing the effects of FA on cancer cell viability, proliferation, cell cycle progression and apoptosis.

Common Name	Cancer Models/Cancer–Cancer Cell Line	Molecular Pathway	Cellular Effect	Ref.
**SFA**				
Myristoleic acid	Prostate—LNCaP cells	Not reported	Cytotoxic effect	[163]
Palmitic acid	Neuroblastoma—SH-SY5Y cells	Reduced insulin-dependent Akt/ERK phosphorylation Up-regulated Toll-like receptor 4 Increased pro-inflammatory cytokines IL6 and TNFα	Impaired insulin signaling Induced inflammation	[164]
	Neuroblastoma—SH-SY5Y and T98G cells	Increased ROS production and lipid peroxidation	Cytotoxic effects Increased oxidative stress Induced apoptosis Increased % of cells in early apoptosis Decrease % cells in G_2_-M phase	[165]
Lauric acid	Endometrial—Ishikawa cells Breast—SkBr3 cells	Triggered EGFR, ERK and c-Jun phosphorylation. Increased c-fos and p21Cip1/WAF1 protein expression Increased ROS generation	Decreased cell viability Increased apoptosis	[166]
	Colorectal—CaCo2 cells	Reduced GSH availability Increased ROS generation	Reduced the cells in G0/G1 and arrested the cells in the S and G2/M phases Induced apoptosis	[167]
**MUFA**				
Elaidic acid	Colorectal—CT26 and HT29 cells	MAPK-Wnt pathway	Increased cell growth, antiapoptotic survival, and invasion	[168]
Trans vaccenic acid—a geometric isomer of oleic acid	Breast—MCF-7 cells	Down-regulated the expression of Bcl-2 procaspase-9	Inhibited cell proliferation	[169]
	Nasopharyngeal—5-8F and CNE-2 cells	Increased p-Akt levels and bad phosphorylation on Ser-136 and Ser-112.	Inhibited cell proliferation in a dose-dependent manner	[170]
	Breast—MDA-MD-231 cells	Binding to GPR40 (coupled to G_i_/G_o_ and G_q)_ → activation of PLC/PKC/Ca^2+^ and PI3K/Akt and MEK1/2/Src pathways	Promoted cell growth and proliferations	[171]
	Renal—786-O cells	Bining to GPR40 → activation of integrin-linked kinase (ILK) → activation of Akt and COX-2	Promoted cell proliferation	[172]
OA	Cervical -HeLa cells	Up-regulated the expression of CD36, a FA transporter + induced Src kinase and downstream ERK1/2 pathway activation in a CD36-dependent manner	Promoted cell proliferation	[173]
	Liver—HepG2 cells	Upregulation of FABP5 and HIF-1α	Improved cell survival	[174]
	Gastric—HGC-27 cells Breast—MDA-MB-231 cells	AMPK activation → increased β-oxidation	Increased cell proliferation and migration	[175]
	Orthotopic pancreatic cancer model —Panc02 cells	Increased expression of epithelial-to-mesenchymal transition factors SNAI-1 (Snail) and Zeb-1	Increased cell proliferation + >2.5-fold increase in cell migration	[176]
	Mice lung tumorigenesis model	Inhibited PGE2 production and inactivated the Erk cascade	Reduced lung tumor incidence and tumor multiplicity (percentage of mice with tumors)	[177]
	Intrasplenic Lewis lung carcinoma—LLC implanted mice + HMVEC cells	Inhibited DNA synthesis in LLC cells but not in HMVEC	Inhibited angiogenesis Inhibited liver metastasis and metastatic tumor growth	[178]
Palmitoleic acid	Lung—A549 cells were SCD activity was inhibited by SCD inhibitor CVT-11127	SCD inhibition blocks cell cycle in G_1_/S phase + reduces lipid synthesis; palmitoleic acid reversed the effects of the blockade	Restored cell cycle progression	[179]
Erucic acid	Glioma—C6 cells	Decreased S-phase inhibitory potency of DOX	Decreased cell proliferation	[180]
**PUFA**				
EPA	Breast—MCF-7 cells	Decreased Akt1 activity, a downstream mediator in PI3K signaling pathway	EPA restored apoptotic response to tamoxifen in hyperactive Akt cells	[181]
	Ovarian tumor rat model	Inhibited protein expression of PI3K/Akt, ERK1/2 and NF-κB P65 Increased protein expression of cytochrome *C* and caspase-3	Anti-tumor activity	[182]
	Breast—4T1 Melanoma—B16F10	Inhibited indoleamine 2,3-dioxygenase 1 (IDO)expression through the suppression of the AKT/mTOR signaling pathway	No cytotoxic effect at the tested doses	[183]
	Ovarian—SKOV3 and ES2 cells Ovarian cancer xenograft models	Inhibited the phosphorylation of AKT and ERK1/2 Activated the GPR30-cAMP– protein kinase A signaling pathway	Inhibited cell proliferation Blocked tumor growth Decreased tumor volume and weight through GPR30	[162]
	Pancreatic—MIA-PaCa-2 and Capan-2 cells	Increased ROS production Induced caspase-8 activation	Increased apoptosis	[184]
	Colorectal—murine C26 cells	Increased caspase 3/7 activity	Decreased cell viability	[185]
	Prostate—PC3 cells	Inhibited proline-rich tyrosine kinase (Pyk)2 and extracellular signal-regulated kinase 1/2 phosphorylation	Decreased cell viability	[186]
	Colorectal—HCT-116 cells Hhypopharyngeal—FaDu cells Cervix—SiHa cells	Induced lipid peroxidation Lack of caspase-3 cleavage → EPA does not induce apoptosis Up-regulation of ferroptosis GPX4	Increased cytotoxic effect Cell death via ferroptosis	[124]
DHA	Pancreatic—MIA-PaCa-2 and Capan-2 cells	Increased ROS production Induced caspase-8 activation	Increased apoptosis	[184]
	Endometrial—RL95-2 cells	Activation of caspase-9, -3, and -7 Activation of caspase-8/BID	DHA alone did not induce apparent cytotoxic effects; 125 μM DHA and 5 μM triacsin C increased the sub-G1 population and apoptotic fragments.	[187]
	Colorectal—HCT-116 cells Hhypopharyngeal—FaDu cells Cervix—SiHa cells	Induced lipid peroxidation Lack of caspase-3 cleavage → DHA does not induce apoptosis Up-regulation of ferroptosis GPX4	Increased citotoxic effect Cell death via ferroptosis	[124]
	Breast—MCF-7 cells	Increased ROS production Activation of caspase88	Induced apoptosis	[188]
	Prostate—PC3 cells DOX resistant	Increased ROS production Down-regulated GSTπ expression Activated PI3K/AKT/Nrf2/GPX4 signaling pathway A	Induced cytoprotective autophagy and ferroptosis	[189]
	Ovarian—Hey and IGROV-1 cells Transgenic mouse model of ovarian cancer	Increased ROS production Down-regulated CDK4, CDK6, Cyclin D1, Ki67 and VEGF expression	Decrease cell viability Inhibited cellular proliferation Induced cell cycle arrest Induced apoptosis	[190]
	Ovarian—A2780 cells	Increased ROS production and caspase-1 activation Led to the loss of mitochondrial membrane potential	Induces pyroptosis and mitochondrial dysfunction Reduced mitochondrial content Decreased cell viability and proliferation Increased membrane permeability and LDH release	[191]
	Triple negative breast—MDA-MB-231 cells	Increased caspase-1and gasdermin D activation Enhanced IL-1β secretion; translocated HMGB1 toward the cytoplasm Formed membrane pores	Triggered pyroptosis cell death	[192]
	Colon—SW620 and Caco-2 cells	Induced nuclear translocation of the oxidative stress sensor NFE2L2 Induced oxidative stress	Colon cancer cells with low basal autophagy are more sensitive to stress and may be targeted by DHA	[193]
	Lung—A549 cells	Increased the accumulation of intracellular ROS Down-regulated the levels of HEF1, MMP 9, and VEGF Inactivated Akt phosphorylation	Inhibited proliferation and induced apoptosis Suppressed the invasion and metastasis	[194]
	Ovarian—PA1 cells Lung—H1299 cells Brain—D54MG cells Cervical—SiHa cells	Increased mitochondrial ROS production → mitochondrial dysfunction Induced activation of MAPKs	Induced apoptosis	[195]
	Promyelocytic leukemia—HL60 cells	Increased cytochrome c release from mitochondria Induced mitochondrial swelling and membrane depolarization	Induced apoptosis via mitochondrial pathway	[196]
Phloridzin docosahexaenoate (PZ-DHA)	Triple negative breast—MDA-MB-231, 4T1 and SUM149 cells	Inhibited the expression of matrix metalloproteinase 2 (MMP2); Inhibited Akt/phosphoinositide 3-kinase and extracellular signal-regulated kinase 1 and 2	Cytotoxic effects Inhibition of proliferation	[197]
13R,20-dihydroxydocosahexaenoic acid (13*R*,20-diHDHA)	Breast—Cancer stem cells (CSCs)	Increased ROS production Reduced the phosphorylation of nuclear signal transducer and activator of transcription 3 (Stat3)	Inhibited mammosphere formation, colony formation, migration, and invasion → inhibited breast cancer stemness	[198]
Lysophosphatidylcholine-DHA	Triple negative breast—MDA-MB-231 cells	Increased oxidative stress Increased membrane cell damage	Cytotoxic effect for glycerophosphocholine-based DHA (LPC-DHA and PC-DHA); MAG-DHA and free DHA were less toxic, while DAG-DHA, TAG-DHA did not exert any cytotoxic effects	[199]
Didocosahexaenoin	Prostate—PC3 cells	Increased ROS production Increase mitochondrial membrane potential Activated caspase-3/7	High selective cytotoxic effects	[200]
DHA-PC, DHA- TG, DHA- ethyl esters (DHA-EE)	Lung—95D cells	DHA-PC and DHA-TG up-regulated the PPARγ and RXRα signal DHA-PC and DHA-TG inhibited the expression of NF-κB and Bcl-2, and enhanced the expression of Bax and caspase-3	DHA-PC and DHA-TG induced cell contraction, increased concentration of cell heterochromatin, vacuolization of cytoplasm, edema of ER and mitochondria and promoted apoptosis DHA-EE had no significant effect on cell viability	[201]
LA	Colorectal—LoVo cells	Increased mitochondrial ROS production decreased cell antioxidant capacity → mitochondrial dysfunction	Induced apoptosis via mitochondrial pathway	[202]
	Colorectal—LoVo and RKO cells	Increased mitocohndrial ROS production → mitochondrial dysfunction Increased intracellular Ca^2+^ accumulation Activated caspase-9 and caspase-3 Decreased ATP level Increased the Bax/Bcl2 ratio	Induced apoptosis via mitochondrial pathway	[203]
Conjugated-LA	Brain—U-87 MG cells, Lung—A549 cells, Breast—MCF-7 cells, Colon—Caco 2 cells, Prostate -PC3 cells, Cervical—HeLa cells	Not reported	Cytotoxic activity on PC-3	[204]
Punicic acid	Hypopharyngeal -FaDu cells Colorectal—HCT-116 celss	Increases lipid peroxydation	Decreased cell viability, cytotoxic effects Induces ferroptosis	[205]
Derivatives of DHA and LA	Breast—MCF-7 cells	Suggested, but not demonstrated: induced oxidative stress, lipid raft disruption, enhanced uptake, and apoptosis activation.	Reduced cancer cell viability Induced necrosis and apoptosis	[57]
Ethyl ester-DHA and -EPA	Multiple myeloma - L363, OPM2, U266 cells	Increased ROS production and oxidative stress	Increased cytotoxic effects Induced necroptosis	[206]

## Data Availability

No new data were created or analyzed in this study.

## Abbreviations

The following abbreviations are used in this manuscript:

AA	Arachidonic acid
ADME	Absorption, distribution, metabolism and excretion
ALA	Alpha-linolenic acid
BA	Betulinic acid
BFA	Branched fatty acid
BSA	Bovine serum albumin
Ce6	Chlorin e6
CLA	Conjugated linoleic acid
COL	Cholesterol
DCC	Dicyclohexylcarbodiimide
DHA	Docosahexaenoic acid
DMAP	4-Dimethylaminopyridine
DMG-PEG2000	Dimyristoyl glycerol polyethylene glycol 2000
DOX	Doxorubicin
DSPC	Distearoylphosphatidylcholine
DSPE	Distearoylphosphatidylethanolamine
EDC	1-Ethyl-3-(3-dimethylaminopropyl)carbodiimide
EPA	Eicosapentaenoic acid
EPR	Enhanced permeability and retention
FA	Fatty acid
FR	Folate receptor
GSH	Glutathione
HSA	Human serum albumin
LAPs	Lipidic prodrugs
LC-FA	Long-chain fatty acid
LCT	Long-chain triglyceride
LDL	Low-density lipoprotein
LNPs	Lipid nanoparticles
MCT	Medium-chain triglyceride
MUFA	Monounsaturated fatty acid
NPs	Nanoparticles
OA	Oleic acid
PC	Phosphatidylcholine
PDI	Polydispersity index
PEG	Polyethylene glycol
PLGA	Poly(lactic-co-glycolic acid)
PS	Phosphatidylserine
PTX	Paclitaxel
PUFA	Polyunsaturated fatty acid
QUE	Quercetin
ROS	Reactive oxygen species
SNEDDS	Self-nanoemulsifying drug delivery systems
SFA	Saturated fatty acid
TME	Tumor microenvironment
T3P	Propylphosphonic anhydride
UFA	Unsaturated fatty acid
5-FU	5-Fluorouracil
